# Ciliary proteins specify the cell inflammatory response by tuning NFκB signalling, independently of primary cilia

**DOI:** 10.1242/jcs.239871

**Published:** 2020-07-08

**Authors:** Megan Mc Fie, Lada Koneva, Isabella Collins, Clarissa R. Coveney, Aisling M. Clube, Anastasios Chanalaris, Tonia L. Vincent, Jelena S. Bezbradica, Stephen N. Sansom, Angus K. T. Wann

**Affiliations:** 1 Kennedy Institute of Rheumatology Research, Nuffield Department of Orthopaedics, Rheumatology and Musculoskeletal Sciences, Medical Sciences Division, University of Oxford, Oxford OX3 7FY, UK; 2 School of Engineering and Materials Science, Queen Mary University of London, Mile End Road, London E1 4NS, UK

**Keywords:** IFT88, Inflammation, Macrophage, NFkB, Primary cilia, TTBK2

## Abstract

Complex inflammatory signalling cascades define the response to tissue injury but also control development and homeostasis, limiting the potential for these pathways to be targeted therapeutically. Primary cilia are subcellular regulators of cellular signalling, controlling how signalling is organized, encoded and, in some instances, driving or influencing pathogenesis. Our previous research revealed that disruption of ciliary intraflagellar transport (IFT), altered the cell response to IL-1β, supporting a putative link emerging between cilia and inflammation. Here, we show that IFT88 depletion affects specific cytokine-regulated behaviours, changing cytosolic NFκB translocation dynamics but leaving MAPK signalling unaffected. RNA-seq analysis indicates that IFT88 regulates one third of the genome-wide targets, including the pro-inflammatory genes *Nos2*, *Il6* and *Tnf*. Through microscopy, we find altered NFκB dynamics are independent of assembly of a ciliary axoneme. Indeed, depletion of IFT88 inhibits inflammatory responses in the non-ciliated macrophage. We propose that ciliary proteins, including IFT88, KIF3A, TTBK2 and NPHP4, act outside of the ciliary axoneme to tune cytoplasmic NFκB signalling and specify the downstream cell response. This is thus a non-canonical function for ciliary proteins in shaping cellular inflammation.

This article has an associated First Person interview with the first author of the paper.

## Abstract (precis)

**Summary:** Ciliary proteins, acting independently of the ciliary axoneme, regulate the dynamics of cytosolic NFκB, but not other signalling pathways, defining an important subset of the inflammatory response.

## INTRODUCTION

Cells and tissues elicit complex, context-dependent and heterogenic responses to pro-inflammatory cues. A wide range of stimuli activates inflammatory signalling, inducing an even broader spectrum of downstream cell and tissue behaviours that underpin important developmental, homeostatic and pathophysiological responses. The nature of these responses is shaped not only by the transcriptional and epigenetic state of the cell involved, but also by the differential recruitment and dynamics of cytosolic signalling pathways (Purvis and Lahav, 2013). This is exemplified by the temporal encoding of the NFκB signalling pathway (Tay et al., 2010). The timing and locations of the lynchpin biochemical events in the NFκB cascade, namely the phosphorylation and destruction of the inhibitory protein IκB and subsequent release and nuclear translocation of the NFκB transcription factors such as P65 (also known as RELA), are likely critical for how cells integrate extracellular cues and elicit an appropriate downstream response (Mitra et al., 2017). Pathway dynamics and amplitudes in both stromal and immune cells are critical in defining tissue inflammation, and thus dissecting the means by which response specificity is encoded, is vital for understanding and therapeutically targeting inflammatory pathology.

In recent decades, the primary or immotile cilium and its associated collec tive of signal transduction machinery or ‘ciliome’, have been actively researched, after being largely ignored for over a century. The most well-known function of the primary cilium is to act as a single, microtubule-based scaffold where the ciliary proteins directly regulate the transduction events associated with ligand-induced Hedgehog (Hh) signalling, thus tuning transcriptional output (Bangs and Anderson, 2017). Canonically, this regulation takes place within the nanoscale axonemal compartment, supported by intraflagellar transport (IFT) powered by kinesins and dyneins. As a result of this regulatory influence over key developmental pathways such as Hh signalling, ciliary function is now recognized to be critical to metazoan life, and congenital dysfunction of cilia/ciliary proteins results in the human ciliopathies (Reiter and Leroux, 2017). The ciliary compartment assembles from the basal body, formed by modification of the mature centriole. Ciliary assembly at the microtubule-organizing centre connects this organelle to the wider cellular transport cytoskeletal system, supporting a role as a transduction hub for cellular signalling. The cilium may be a critical component of a conserved cytosolic–nuclear pathway, enabling signal integration (Satir and Satir, 2019). Importantly, there are documented non-ciliary roles for ciliary proteins (Delaval et al., 2011), including IFT proteins, in cell types that do not assemble cilia such as immune cells (Finetti et al., 2009; Stinchcombe and Griffiths, 2014).

We previously identified that a hypomorphic mutation affecting the ciliary protein intraflagellar transport protein 88 (IFT88) inhibits the cellular response to the inflammatory cytokine interleukin-1 (IL-1), acting below receptor level, at the level of cytosolic IκB kinase (IKK) (Wann and Knight, 2012; Wann et al., 2014). This in turn alters ciliary structure and trafficking, over a period of hours, and, in turn, affects its function (Wann et al., 2013; Thompson et al., 2015). Subsequent work by others *in vitro* in other cell types (Baek et al., 2017), in the contexts of established ciliopathies (Viau et al., 2018; Zimmerman et al., 2018; Zimmerman et al., 2019) and previously ciliary-unrelated disease (Dinsmore and Reiter, 2016), supports a putative link between the cilium and inflammatory signalling.

However, it remains unclear whether this really represents a role for the primary cilium in transducing inflammatory signalling. The scope of this influence beyond NFκB signalling and over broader transcriptional and cell behaviour, is also unknown. Here, we define the influence of IFT88 in the specification of the cell response to inflammatory cues and explore the role of the cilium. Our data suggest the regulatory influence of IFT88 is specific to a subset of highly influential inflammatory responses through a bias towards regulation of NFκB signal dynamics over other pathways. This regulation is apparently independent of the ciliary axoneme and extends to macrophages.

## RESULTS

### Disruption of IFT88 alters some, but not all, cellular responses to the inflammatory cytokines IL-1β and TNF

**Fig. 1.**
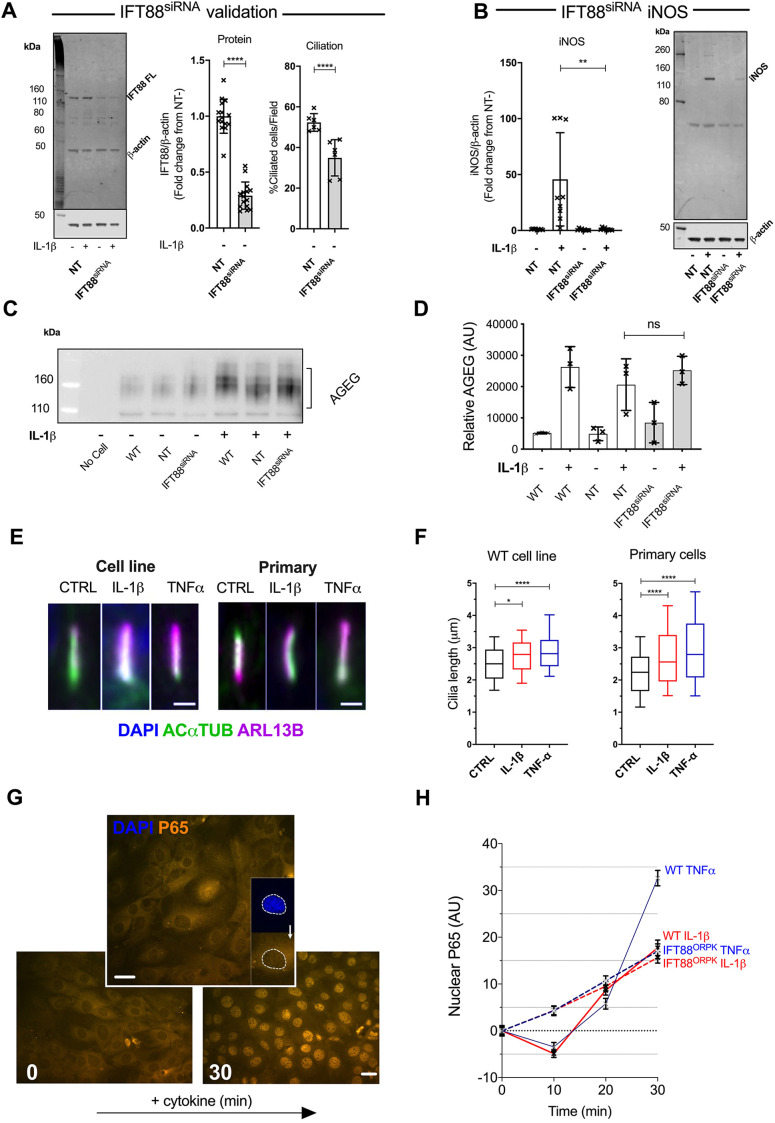
**Hypomorphic and siRNA-mediated disruption of IFT88 in fibroblast-like chondrocytes inhibits select responses to inflammatory cytokines and alters NFκB activation dynamics before ciliary elongation.** (A) Western blot analysis probing for IFT88 (left) in non-targeting (NT) control and IFT88^siRNA^ cell lysates, from cells cultured with or without 10 ng/ml IL-1β for 24 h. IFT88 was quantified (middle) and the data presented as a mean±s.d. fold change from mean IFT88/β-actin levels of the no IL-1β NT condition (set at 1) (*n*=15). ****P<0.0001 (Student's *t*-test. *t*=14 d.f.=28). Effect of siRNA-mediated IFT88 depletion on cilia (right), shown as mean±s.d. percentage ciliated cells in NT and IFT88^siRNA^ cell cultures (*n*=6 fields from two repeats). ****P<0.0001 (Fisher's exact test on contingency data). (B) iNOS protein expression in NT and IFT88^siRNA^ cells cultured with or without 10 ng/ml IL-1β for 24 h. Protein levels were quantified and the data presented as a mean±s.d. fold change from mean iNOS/β-actin levels of the no IL-1β NT condition (set at 1). (*n*=9). **P<0.0001 (Student's *t*-test. *t*=3.215. d.f.=16. Mann–Whitney test. U=18). (C) Western blot analysis of conditioned medium from WT, NT and IFT88^siRNA^ cells cultured with or without 10 ng/ml IL-1β for 24 h, probing for AGEG, with a medium only/no cell control in the first lane. (D) Quantification of the mean±s.d. amount of AGEG in the conditioned medium from WT, NT and IFT88^siRNA^ cells cultured with or without 10 ng/ml IL-1β for 24 h (*n*=3 for each condition). ns, not significant between conditions (*P*=0.1625, two-way ANOVA). (E) Representative images used to calculate cilia length for the WT cell line (left), and primary porcine chondrocytes (right) with or without 10 ng/ml IL-1β or TNF for 24 h. Scale bars: 1 μm. (F) Data from E presented as box-and-whisker plots, where the box represents the 25–75th percentiles, and the median is indicated. The whiskers show the 10–90th percentiles (*n*>90 cilia for each condition for the WT cell line; *n*>260 cilia for each condition for the primary cells). *P=0.0163, ****P<0.0001 (one-way ANOVA with Tukey's multiple comparisons test, WT cell line); ****P<0.0001 (one-way ANOVA with Dunn's multiple comparisons test, primary cells). (G) Representative image of P65 localization in unstimulated (lower left) and 30 min cytokine-stimulated (lower right) WT chondrocytes. Nuclear P65 intensity was quantified by defining the nucleus as a region of interest using DAPI signal (top), and measuring the P65 signal within that region. Scale bars: 5 µm. (H) Data from G presented as mean±s.e.m. for WT (solid lines) and IFT88^ORPK^ (dashed lines) cells, normalized to their own 0 h starting intensity. Experiments had a 30 min time course of 10 ng/ml IL-1β (red lines) or TNF (blue lines), with 10, 20 and 30 min cytokine time points and an unstimulated medium only 0 h time point (*n*=140 to 200 nuclei per condition).

IFT88 was first identified in mammalian cells following an insertional mutation in the non-coding region as part of a random mutagenesis screen, which resulted in a polycystic kidney phenotype (the ‘ORPK’ mouse) (Moyer et al., 1994). Previously, we have employed immortalized fibroblast-like chondrocytes originally isolated from the ORPK mouse. In accordance with previous findings, implicating IFT88 and the primary cilium in IKK activity within the NFκB cascade, (Wann et al., 2014), we found that cells harbouring the truncated IFT88*^ORPK^* hypomorphic protein (Fig. S1A) showed reduced mRNA induction of nitric oxide synthase (iNOS; gene *Nos2*) and prostaglandin E2 synthase (COX2; gene *Ptgs2*), associated with inhibited protein induction as shown previously, following stimulation with IL-1β (Fig. S1B). The IFT88*^ORPK^* mutation is associated with near ablation of cilia assembly, with ∼10% of cells assembling a stunted cilium in this line (Wann et al., 2012). However, IFT88 perturbation also has non-ciliary effects, such as in mitotic cells during the phase of the cell cycle when the cilium is disassembled (Delaval et al., 2011). In light of this, we conducted experiments using the same wild-type (WT) immortalized line as previously to test for the role of IFT88 in IL-1β and TNF responses, using a SMARTpool 4× siRNA approach (IFT88^siRNA^), which depleted IFT88 (Fig. 1A) in subconfluent cells, prior to entry into G0 phase and subsequent ciliogenesis. This was compared against a non-targeting (NT) siRNA pool and resulted in an approximate halving of cilia prevalence from a median of 50.1% down to 27.5% (Fig. 1A). In a similar manner to that seen in IFT88*^ORPK^*, siRNA depletion of IFT88 also inhibited the induction of iNOS (Fig. 1B) and COX2 (Fig. S1D) in response to IL-1β. Depletion of IFT88 also inhibited the response to TNF (Fig. S1D). IFT88 itself was not regulated by IL-1β (Fig. S1E).

Inflammation often induces tissue remodelling. For example pro-inflammatory cytokines, such as IL-1β, increase proteoglycan matrix degradation in cartilage. By co-culturing cells with a recombinant matrix substrate, in this case aggrecan, as described previously (Ismail et al., 2015), cytokine-induced protease activity can be monitored by antibodies against the matrix neoepitopes generated by catabolism (Fig. 1C, quantified in 1D). In contrast to classical inflammatory gene inductions, IL-1β-stimulated catabolism was unaffected by IFT88 depletion. Additionally, IL-1β-mediated transcriptional induction of the gelatinase *Mmp3* and collagenase *Mmp13* were not inhibited by the hypomorphic IFT88*^ORPK^* mutation (Fig. S1F). Thus, not all cytokine-induced responses appear to be IFT88 regulated.

### Targeting IFT88 in fibroblast-like chondrocytes alters NFκB activation dynamics downstream of IL-1β and TNF

*Nos2*, *Ptgs2*, *Mmp3*, *Mmp13* and the proteases responsible for aggrecanolysis have all previously been shown to be transcriptionally dependent on NFκB (Fan et al., 2006; Kobayashi et al., 2013). However, matrix metalloproteinase (MMP) induction and aggrecanase activity, which are apparently unaffected by IFT88 targeting, are often induced later than *Nos2* and *Ptgs2*. Thus, the selectivity of IFT88 regulation, over responses with different timings, led us to explore signalling dynamics downstream of cytokine exposure. While primary cilia in this fibroblast-like chondrocyte line elongate in response to IL-1β or TNF, as seen in primary cells (Wann and Knight, 2012; Wann et al., 2013), elongation takes place hours after IL-1β or TNF exposure (Fig. 1E,F), by which time early responses such as *Nos2* induction are well underway. Having previously investigated IKK activation, IκB phosphorylation and degradation (Wann et al., 2014), we chose here to carefully investigate the cytosolic dynamics of NFκB signaling activation and transduction upstream to transcriptional induction, but downstream to IKK. The NFκB transcription factor P65 is shuttled to the nucleus (Fig. 1G) upon its release from IκB. We used microscopy to monitor and quantify rapid P65 shuttling to the nucleus between 10 and 30 min after IL-1β and TNF stimulation. There was no robust difference in either apparent nuclear P65 observed at baseline (Fig. S2A) or total P65 expression (Fig. S2B). However, IFT88 disruption altered the dynamics of signalling (dashed lines, Fig. 1H; Fig. S2A), most notably decreasing the rate of rapid nuclear accumulations seen between 10 and 30 min, although not always the final amount of nuclear P65 accumulated after 30 min. The mean rates (slope) of translocation between 10 and 30 min for WT cells were 1.15±0.08 and 1.79±0.09 for IL-1β and TNF, respectively. In IFT88*^ORPK^* cells, these rates were reduced to 0.56±0.07 and 0.64±0.09, respectively, both rates statistically significantly different from respective WT rates (mean±s.e.m., *P*≤0.0001 for both cytokines, linear regression analysis). In agreement with previous studies exploring the IKK–IκB axis (Wann et al., 2014), depletion of IFT88 affected the IκB degradation rate albeit, in contrast to IFT88*^ORPK^*, due to a change in absolute starting amounts of IκB (Fig. S2C,D). This led us to hypothesize that IFT88 either regulates the dynamics of NFκB signalling, affecting specific targets on the basis of their transcriptional timing (early or late response), or that, for some responses, the effect on NFκB signalling is compensated for by another, unaffected, pathway. A likely candidate would be the JNK pathway, as it has been shown to be responsible for cytokine-induced protease activity (Ismail et al., 2015).

### Depletion of IFT88 alters one third of the genome-wide IL-1β-induced transcriptional signature independently of transcriptional timing

**Fig. 2.**
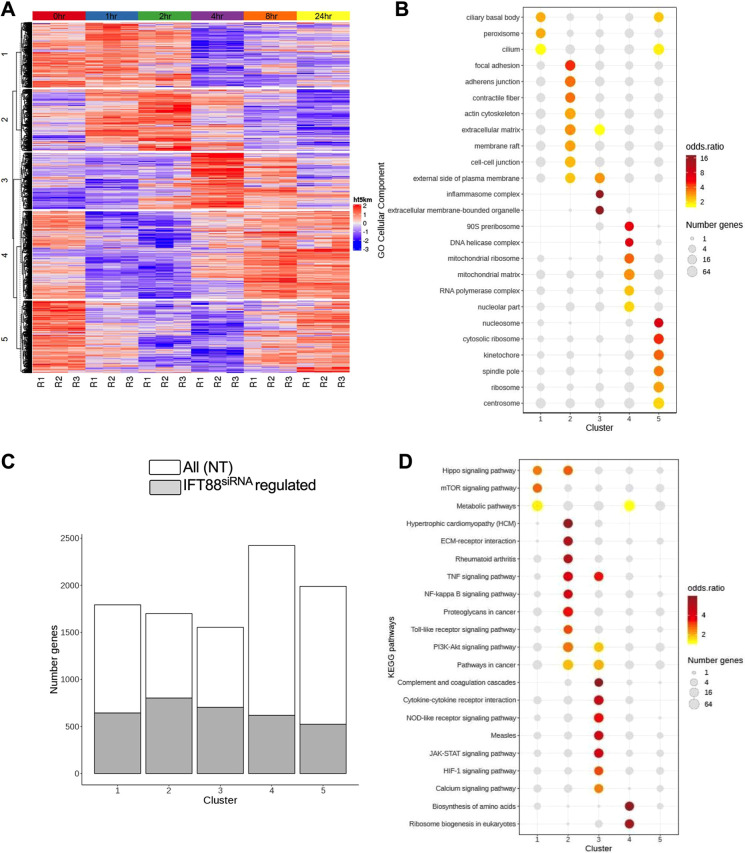
**The IFT88-regulated IL-1β response signature in fibroblast-like chondrocytes is not delineated by transcriptional dynamics.** Non-targeting pool (NT) and IFT88^siRNA^ cells were treated with 10 ng/ml IL-1β for 1, 2, 4, 8 and 24 h with a medium only 0 h control. (A) K-means clustering analysis identified five clusters of significantly IL-1β-regulated genes that showed distinct temporal profiles over the 24 h time course, in the NT control samples (BH adjusted *P*<0.05; fold change >1.5; ImpulseDE2 case-only analysis). There are three replicates per time point and six time points, indicated by the coloured bar at the top of the heat map [0 h (red), 1 h (blue), 2 h (green), 4 h (purple), 8 h (orange) and 24 h (yellow)]. Key, red indicates upregulation of the gene and blue indicates downregulation of the gene with respect to the row mean of the particular gene (across all time points). (B) Over-representation analysis of gene ontology (GO) cellular component categories within each of the clusters identified in A. Significant enrichment (one-sided Fisher's exact tests; BH adjusted *P*<0.05) are in colour; non-significant enrichments are shown in grey. (C) The bar graph shows the numbers of genes falling within each of the clusters shown in A. The fraction of those genes modulated by IFT88 is indicated in grey (BH adjusted *P*<0.05; fold change >1.5; ImpluseDE2 case-control analysis). (D) Over-representation analysis of KEGG pathways amongst the genes within each of the IL-1β induced clusters (as in A) that show a modified response following IFT88^SIRNA^ depletion (coloured grey in C). Significant enrichments (one-sided Fisher's exact tests; BH adjusted *P*<0.05) are coloured; non-significant enrichments are shown in grey.

To further delineate the regulatory effect of IFT88 in regulating the response to pro-inflammatory cytokines, we used RNA sequencing (RNA-seq) to study the impact of IFT88 depletion on the transcriptional response to stimulation with IL-1β. To do so, we profiled IFT88 siRNA-depleted and NT control samples at steady state and at 1, 2, 4, 8 and 24 h following stimulation with IL-1β. To investigate the transcriptional timing hypothesis, we first characterized the normal IL-1β response, separating significantly regulated genes into five clusters according to their temporal profiles (Fig. 2A; Fig. S3A,B). The first cluster identified by this analysis (cluster 1) comprised genes that were reduced in abundance 4–24 h after stimulation. Two sets of genes (clusters 2 and 3) showed a transient increase at 2 h and 4 h, respectively. Cluster 4 comprised a set of genes that was initially reduced before being increased at the final two time points. Finally, we found cluster 5 to contain genes that showed a transient reduction at 1–8 h. Geneset over-representation analysis revealed that genes associated with the cilium and ciliary basal body were significantly enriched in the clusters defined by transient or late reduction in expression (clusters 5 and 1). The transiently reduced cluster was also over-represented for factors associated with the basal body/centrosome, and included the basal body and ciliary-associated genes *Ttbk2* and *Nphp4* (Fig. 2B). Genes that were transiently increased at 4 h (cluster 3) included those associated with the ‘inflammasome complex’ and the pro-inflammatory factor *Nos2*. Case-control analysis of the time course with ImpulseDE2 (Fischer et al., 2018) revealed that while IFT88 depletion affected the expression of genes in all the identified clusters (Fig. 2C; Fig. S3B), altering approximately one-third of the total (Fig. S3C), it did not induce consistent changes in the shape, magnitude or timing of the response signature (Fig. 2C; Fig. S3A). The numbers of genes (*n*=4112) that showed modified expression following IFT88 depletion after IL-1β stimulation greatly exceeded the number that showed changes at baseline (*n*=288 genes, Fig. S3D). Per-cluster KEGG pathway over-representation analysis (See Materials and Methods; Croft et al., 2019) of genes whose expression was modulated by IFT88 depletion, identified changes to NFκB signalling (across a number of clusters), to other pro-inflammatory signalling pathways as well as enrichments for pathways associated with cancer, rheumatoid arthritis, cardiomyopathy, and processes such as ribosome biogenesis, amino acid biosynthesis and complement and coagulation (Fig. 2D).

### IFT88 has a regulatory bias towards NFκB signalling, promoting expression of a subset of important pro-inflammatory genes following IL-1β stimulation

**Fig. 3.**
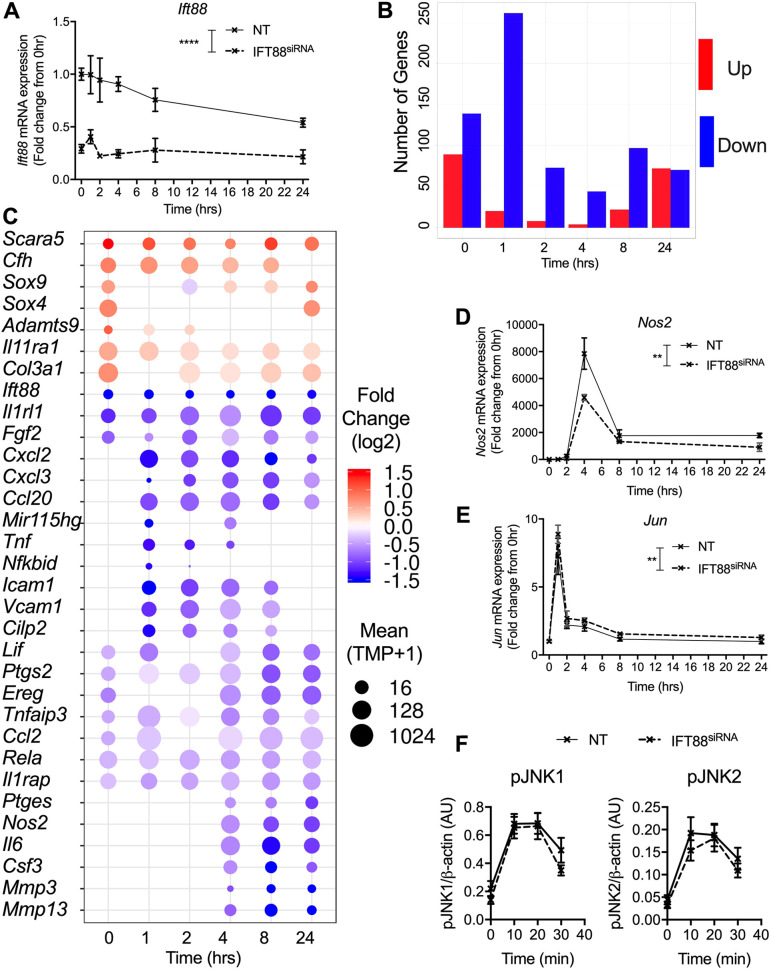
**IFT88 regulates a subset of inflammatory genes in fibroblast-like chondrocytes through regulatory bias towards NFκB.** (A) qPCR quantification of mean±s.d. *Ift88* mRNA expression in non-targeting pool (NT; solid line) and IFT88^siRNA^ (dashed line) cells cultured with 10 ng/ml IL-1β for a time course of 1, 2, 4, 8 and 24 h, presented as a fold change from their respective 0 h unstimulated controls (*n*=3 for each condition). **P<0.01, ****P<0.0001 (two-way ANOVA). (B) Bar graph of the number of significantly (BH-adjusted *P*<0.05; fold change >1.5; DESeq2 analysis) differentially regulated genes that are up- (red) or down- (blue) regulated in the IFT88^siRNA^ condition, with respect to the NT control for each time point. (C) Dot plot of selected genes that are significantly regulated by IFT88 at one or more time points of the 24 h time course (BH adjusted *P*<0.05). (D,E) Quantification of *Nos2* and *Jun* mRNA expression as described for A. (F) Phosphorylated (p)JNK1 (left) and pJNK2 (right) levels in NT and IFT88^siRNA^ cells cultured with or without 10 ng/ml IL-1β for 0 min (NT and IFT88^siRNA^, *n*=8), 10 min (NT and IFT88^siRNA^, *n*=8), 20 min (NT, *n*=6, and IFT88^siRNA^, *n*=5) and 30 min (NT and IFT88, *n*=8). Graphs in F presented as mean±s.e.m. (not significant, two-way ANOVA).

**Fig. 4.**
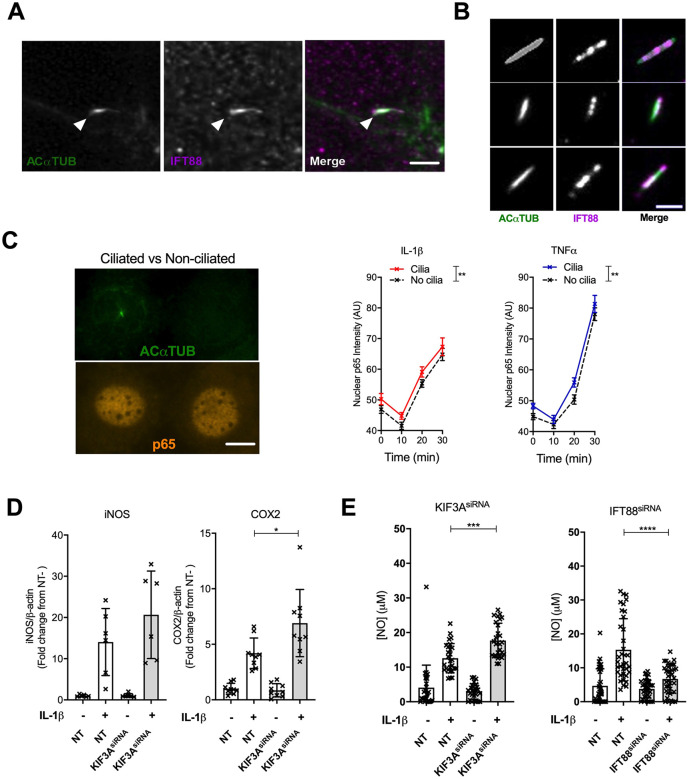
**IFT88 is acting independent to the ciliary axoneme in fibroblast-like chondrocytes.** (A) Confocal immunofluorescence microscopy of chondrocytes, for acetylated α-tubulin (ACαTUB; green), IFT88 (purple) and primary cilia (highlighted by arrowheads). Scale bar: 2 µm. (B) TIRF microscopy of cilia, ACαTUB (green) and IFT88 (purple). Scale bar: 2 µm. (C) NFκB P65 (orange) in a ciliated (top image) and a non-ciliated (bottom image) cell (cilia identifiable by ACαTUB staining in green), 30 min after cytokine stimulation. Scale bar: 5 µm. Graphs on the right show the mean±s.e.m. nuclear P65 fluorescent intensity over a 30 min time course for ciliated (solid) and non-ciliated (dashed) cells in response to either 10 ng/ml IL-1β (red) or TNF (blue, *n*>50 nuclei per condition, per time point). **P=0.005 and 0.002 for IL-1β and TNF, respectively (two-way ANOVA). (D) KIF3A^siRNA^ cells cultured with or without 10 ng/ml IL-1β for 24 h. Protein levels quantified from western blot analysis (Fig. S5B,C) and the data presented as a mean±s.d. fold change from mean iNOS/β-actin or COX2/β-actin levels for the no IL-1β NT condition (iNOS *n*=6; COX2 *n*=9). Not significant (*P*>0.05, Student's *t*-test) for iNOS; *P=0.0188 for COX2 (Mann–Whitney test. *U*=14). (E) Nitric oxide (NO) analysis (mean±s.d.) of conditioned medium from KIF3A^siRNA^ and IFT88^siRNA^ cells cultured with or without 10 ng/ml IL–1β for 24 h compared with NT (*n*=36 and 27, respectively). ***P=0.002, ****P<0.0001 (Mann–Whitney test).

Depletion of IFT88, by siRNA, was validated by quantitative PCR (qPCR) across the time-course in the samples used for the RNA-seq (Fig. 3A). In complement to the global time-course analysis, we also performed differential expression analyses between control siRNA and IFT88-depleted cells at each of the individual time-points (Fig. S4A–E). These individual analyses revealed that depletion of IFT88 tended to result in downregulation of genes relative to control at 1–8 h post stimulation (Fig. 3B). Genes that showed a significantly lower expression included pro-inflammatory factors such as *Tnf*, *Il6*, *Nos2* (iNOS), *Ptges*, *Csf3*, *Ccl2* and *Ptgs2* (COX2), and growth factors, as well as adhesion and matrix-related molecules (Fig. 3C). Reduced expression of *Il1rap* prior to challenge with IL-1β might have contributed to the weakened inflammatory response of the IFT88^siRNA^ cells. Interestingly, genes increased by IFT88 depletion in the absence of IL-1β stimulation (0 h) included *Sox9* and *Sox4* genes, which have established roles in promoting chondrogenesis (Akiyama et al., 2002; Zhang et al., 2015), and together with reduced expression of *Lif*, indicates that loss of IFT88 may favour chondrocyte differentiation. We confirmed marked reductions in *Nos2* induction (Fig. 3D) and *Ptgs2* (Fig. S4I) by qPCR. In contrast with results from IFT88*^ORPK^*, *Mmp3* and *Mmp13* were also inhibited by siRNA depletion of *IFT88* (Fig. 3C; qPCR validated in Fig. S4J,K). The selectivity of the effect of IFT88 depletion on the expression of NFκB-regulated genes was maintained. For example, the induction of *Jun* expression, an indicator of JNK pathway signalling, was not inhibited, but slightly enhanced over the time-course (Fig. 4E). In further support of the idea that other signalling pathways might compensate for the changes in the NFκB pathway with depletion of IFT88, activation of the JNK and ERK pathways was unaffected by IFT88 depletion (Fig. 3F; Fig. S4F–H). Thus the regulatory specificity is due to NFκB pathway bias over other pathways activated by cytokine, rather than timing of the cascade. To further understand the IFT88-mediated regulation, we next focused on the location of the putative interaction between the ciliary protein IFT88 and the NFκB-dependent inflammatory response, to address whether this takes place in the cilium.

### IFT88 regulation of cytokine-induced signalling is independent of the ciliary axoneme

To further explore the molecular nature of IFT88-mediated regulation of cytokine response via NFκB, we first assessed the subcellular location of IFT88 using confocal (Fig. 4A) and TIRF microscopy (Fig. 4B). Both confocal and TIRFM images confirmed that IFT88 staining was concentrated in small, punctate signals along the length of the cilium and often accumulated at the base (Fig. 4A, arrowhead) and tip. However, confocal microscopy indicated IFT88 expression was not exclusive to the ciliary axoneme. Returning to P65 shuttling analysis on a cell-by-cell basis, we analysed shuttling in quiescent WT populations with and without an identifiable ciliary axoneme, as indicated by acetylated α-tubulin staining (Fig. 4C, image panels). When quantified, no differences were seen between the shuttling dynamics of ciliated and non-ciliated cells (slope values for IL-1β experiment: WT ciliated 1.16±0.13, non-ciliated 1.17±0.11, *P*=0.95, linear regression analysis 10–30 min; slope values for TNF experiment: WT ciliated 1.85±0.13, non-ciliated 1.78±0.12, *P*=0.72, linear regression analysis 10–30 min; mean±s.e.m.). While levels of P65 did appear slightly lower across both cytokine time-courses in non-ciliated cells (Fig. 4C), these data imply that alterations to dynamics, such as those seen with IFT88 perturbation (Fig. 1F), were not associated with the ciliary axoneme, despite the later axonemal elongation. Next, we depleted KIF3A (Fig. S5A), a kinesin required for anterograde axonemal (IFT) transport in cilia, which reduced cilia prevalence by half. Strikingly, where IFT88 depletion inhibited the response, a comparable depletion of KIF3A, which also had a similar effect on ciliation (Fig. S5A), resulted in a small enhancement of iNOS induction (Fig. 4D; blot in Fig. S5B) and COX2 (Fig. 4D; Fig. S5C). KIF3A depletion raised IFT88 expression (Fig. S5D) and cytokine reduced KIF3A expression (Fig. S5E) implying an interaction at expression level between KIF3A and IFT88. NFκB P65 shuttling has been demonstrated to be dynactin/cytoplasmic dynein-dependent (Shrum et al., 2009), but inhibition of type II cytoplasmic (ciliary) dynein, associated with ciliary retrograde trafficking, by treatment with ciliobrevin (Firestone et al., 2012) had no observed effect on P65 shuttling (Fig. S5F). These data imply that the roles of IFT88 and KIF3A in regulation of the inflammatory response and the IFT88-mediated regulation of P65 translocation are not dependent on axoneme activities.

### Non-ciliated macrophages express IFT88

**Fig. 5.**
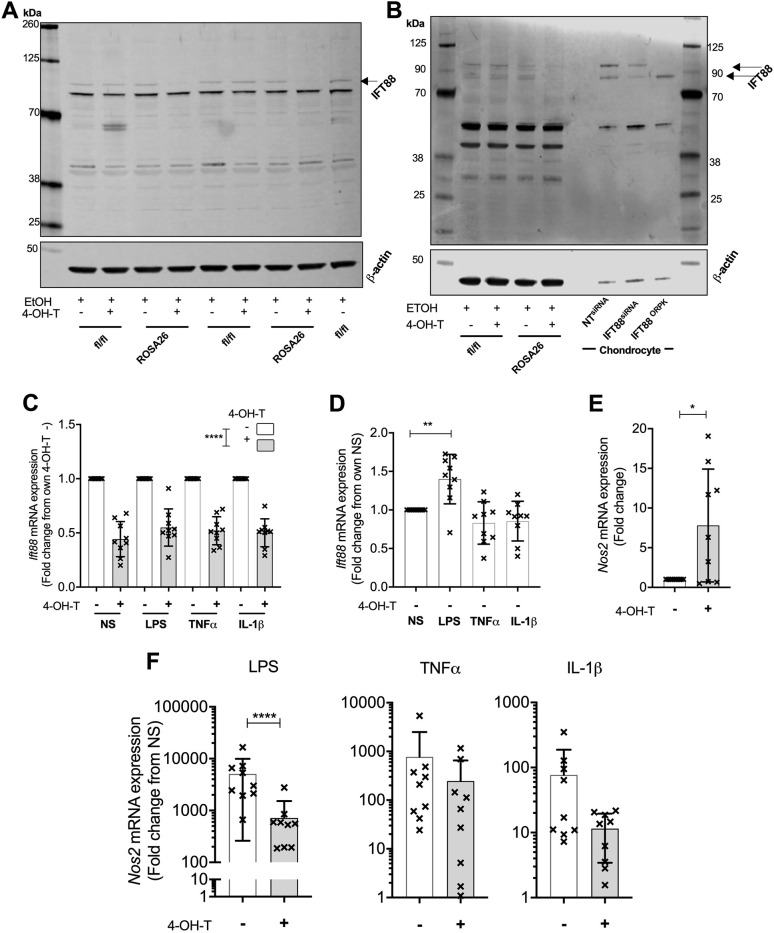
**IFT88 is expressed at low levels in non-ciliated bone marrow-derived macrophages, where its removal inhibits inflammatory response.** (A) Western blot analysis of IFT88 in BMDMs from either IFT88*^fl/fl^* control or IFT88*^fl/fl^ /*ROSA26ERT^2^ mice, all treated with the ethanol vehicle with or without 4-hydroxytamoxifen (4-OH-T). β-actin was used as a loading control. Cells cultured for 7 days after isolation before lysate collection. (B) Western blot analysis using alternative IFT88 antibody in BMDMs from either IFT88*^fl/fl^* control or IFT88*^fl/fl^ /*ROSA26ERT^2^ mice, all treated with the ethanol vehicle with or without 4-OH-T. β-actin was usedas a loading control. Cells cultured for 7 days after isolation before lysate collection. Blot also shows expression in chondrocyte line with and without siRNA to IFT88 and the ORPK mutation (note less protein was loaded for these samples). (C) qPCR analysis probing for *Ift88* in mRNA from BMDMs treated with or without 4-OH-T and stimulated as indicated of not stimulated (NS). Data presented as mean±s.d. mRNA expression as a fold change from the paired negative 4-OH-T (*n*=9). ****P<0.0001 (two-way ANOVA). (D) *Ift88* mRNA expression in all stimulation conditions at point of stimulation shown as mean±s.d. (*n*=9). **P<0.01 (one-way ANOVA). (E) qPCR analysis probing for *Nos2* in mRNA from BMDMs treated with or without 4-OH-T. Data presented as mean±s.d. mRNA expression as a fold change from the paired negative 4-OH-T condition (*n*=9). *P=0.0110 (Student's *t*-test, t=2.873 d.f.=16). (F) qPCR analysis probing for *Nos2* in mRNA from BMDMs treated with or without 4-OH-T, stimulated with LPS (left), TNF (middle) and IL-1β (right). Data presented as mean±s.d. mRNA expression as a fold change from the respective with or without 4-OH-T paired NS condition (*n*=9). ****P<0.0001 (two-way ANOVA).

In light of the lack of apparent role for the axoneme on NFκB dynamics in ciliated cells, we investigated whether IFT88-mediated regulation of the response to cytokine and lipopolysaccharide (LPS) occurs in macrophages, which do not assemble primary cilia (Zimmerman et al., 2019). Bone marrow-derived macrophages (BMDM) isolated from both IFT88^fl/fl^ control and IFT88*^fl/fl^*/ROSA26CreER^T2^ expressed *Ift88* mRNA and protein (Fig. 5A, top band IFT88 at ∼94 kDa) at a readily detectable level, and the protein could be deleted through 4-hydroxytamoxifen treatment *in vitro* in IFT88*^fl/fl^*/ROSA26CreER^T2^ cells as expected (Fig. 5A). In order to confirm this, we tested for IFT88 expression, using an alternative antibody, in macrophage samples with and without Cre-mediated excision, and in chondrocyte samples, with and without siRNA targeting of IFT88 and in samples harbouring the ORPK mutation (Fig. 5B). This confirmed low expression, relative to chondrocyte, and this time two 4-hydroxytamoxifen-sensitive bands were identified, in macrophages and chondrocytes, the largest of which was missing in ORPK samples, and both were reduced in fibroblasts targeted with siRNA. In line with loss of protein, *Ift88* mRNA expression was reduced by tamoxifen treatment *in vitro* in all experimental conditions in macrophages isolated from IFT88*^fl/fl^*/ROSA26CreER^T2^ mice (Fig. 5C). IFT88 expression was unaffected by exposure to TNF or IL-1β, but increased by LPS treatment (Fig. 5D). At baseline, a statistically significant, but highly varied, increase in *Nos2* expression was observed with 4-hydroxytamoxifen treatment compared with control (Fig. 5E). Regardless, LPS, TNF and IL-1β all produced a significant increase in expression of *Nos2* as indicated by qPCR analysis at 24 h post stimulation. In all three pro-inflammatory conditions, IFT88 deletion resulted in reduced induction of *Nos2* (Fig. 5F) when normalized to its own baseline *Nos2* levels*.* In LPS stimulation experiments, IFT88 deletion resulted in a mean fold induction of expression of 719.9±805.4 compared with 5094±4832 in controls (mean±s.d.). In response to TNF the IFT88-deleted mean fold induction was 246.9±407.6 compared with 772.1±1737 in control cells. For IL-1β experiments the mean fold change in *Nos2* expression relative to unstimulated vehicle only controls was 11.49±8.402 in IFT88-deleted cells compared with 76.91±111.2 in controls. Thus, these data show that there is also an inhibition of *Nos2* induction in response to inflammatory cues when IFT88 is deleted in non-ciliated macrophages. Collectively with microscopy and KIF3A depletion experiments, these data in both ciliated and non-ciliated cells suggest an axoneme-independent mechanism for IFT88-mediated regulation of the response to inflammatory cues.

### Multiple ciliary proteins regulate the cell response to cytokine upstream of the basal body/centrosome

**Fig. 6.**
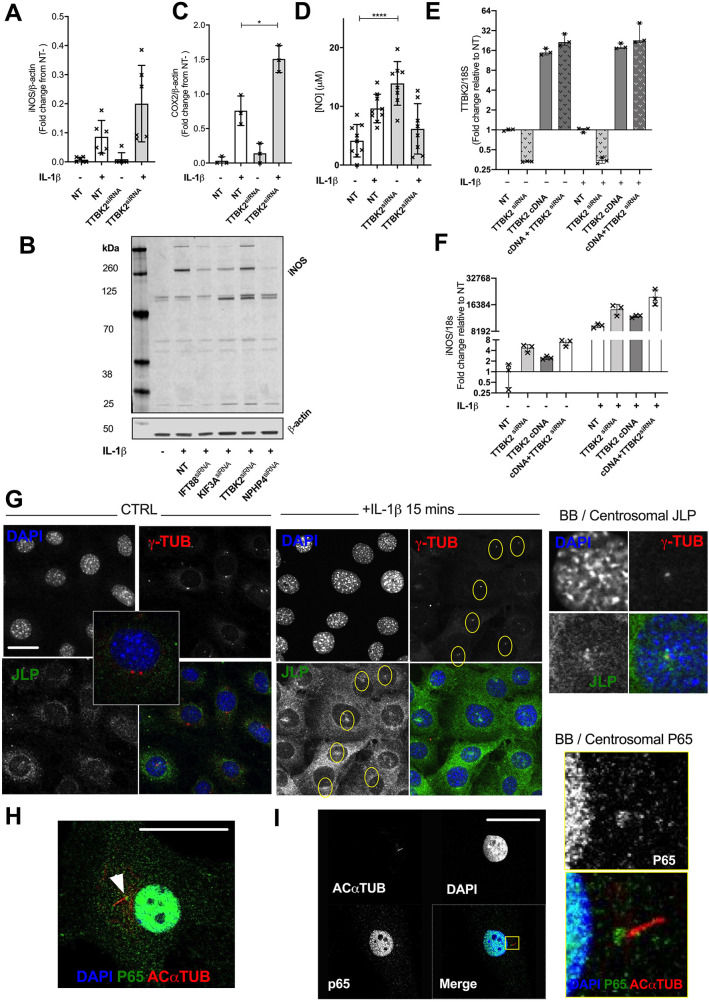
**Regulation of the response to cytokine in fibroblast-like chondrocytes by TTBK2.** (A) iNOS and (C) COX2 protein expression in non-targeting pool (NT) and TTBK2^siRNA^ cells cultured with or without 10 ng/ml IL-1β for 24 h. Protein levels were quantified from western blot (representative blots shown in Fig. S6B,C, respectively) and the data presented as a mean±s.d. fold change from mean iNOS/β-actin or COX2/β-actin levels of the no IL-1β NT condition (iNOS *n*=6; COX2, *n*=3). Not significant (*P*>0.05, Student's *t*-test) for iNOS; *P=0.0110 (Student's *t*-test. *t*=4.483 d.f.=4) for COX2. (B) Western blot analysis of iNOS in NT cells and cells with siRNA depletion of IFT88, KIF3A, TTBK2 and NPHP4, with β-actin as a loading control, with or without 10 ng/ml IL-1β for 24 h. (D) Nitric oxide (NO) analysis of conditioned medium from TTBK2^siRNA^ cells cultured with or without 10 ng/ml IL–1β for 24 h compared with NT (*n*=9, respectively) presented as mean±s.d. [*n*=8 (NT), 9 (TTBK2 siRNA)]. ****P<0.0001 (Mann–Whitney test). (E,F) qPCR analysis of TTBK2 (E) and iNOS (F) relative to NT condition (*n*=3) in TTBK2 siRNA, TTBK2 overexpression (cDNA) and both conditions. Graphs presented as mean±s.d. (G) Confocal immunofluorescence microscopy of control and cytokine-exposed WT cells, labelled with DAPI (blue), and stained for JLP (green) and γ-tubulin (γ-TUB; red), illustrating basal body (BB)/centrosomal localization (yellow ovals) of JLP upon cytokine exposure. (H,I) Confocal immunofluorescence microscopy of a cytokine-exposed WT chondrocyte, labelled with DAPI (blue), and for P65 (green) and acetylated α-tubulin (ACαTUB; red), illustrating P65 localization to the basal end of the cilium. Scale bars: 10 μm.

Our RNA-seq dataset identified an enrichment of basal body/centrosome genes in the response to IL-1β (Fig. 2B) including the ciliogenesis kinase TTBK2, which uncaps the basal body enabling axonemal extension (Cajanek and Nigg, 2014) and the basal body/transition zone protein NPHP4 (Mollet et al., 2005; Arts et al., 2007). Targeting TTBK2 by siRNA (validated by qPCR, Fig. S6A) did not inhibit COX2 and iNOS expression, but, as seen for KIF3A depletion, even increased the response (Fig. 6A,B; blots Fig. S6B,C). In contrast to KIF3A, TTBK2 depletion had no effect on IFT88 expression (Fig. S6D) suggesting this effect was not simply the result of ciliome protein expression interactions but rather that these ciliary proteins might have a complex, interactive and regulatory roles at the basal body or elsewhere in the cytosol, instead of at the ciliary axoneme. Measuring levels of downstream NO released into the culture medium, we observed an increase upon depletion of TTBK2 even without cytokine treatment (Fig. 6D). A comparative analysis of iNOS induction revealed that depletion of NPHP4 (validated by qPCR, Fig. S6E) reduced the response to cytokine in a similar manner to depletion of IFT88 (Fig. 6B), in contrast to the enhancing effects of depletion of KIF3A and TTBK2. To explore further the role of TTBK2 in inflammatory signalling, we overexpressed TTBK2 both without and with siRNA depletion (Fig. 6E). Both TTBK2 depletion and overexpression without siRNA, increased basal iNOS expression and further enhanced the induction of iNOS by cytokine (Fig. 6F) implicating TTBK2 in complex regulation of iNOS expression. Collectively, these four ciliome components regulate the cell response to cytokine, albeit in opposite directions. In our experiments that assessed P65 shuttling in chondrocytes, we often observed P65 accumulation at the base of the cilium by confocal imaging (arrowhead Fig. 6H,I). This accumulation, only visible towards the end of translocation (after 25 min), is likely a consequence of P65 reaching this location following dynein-dependent shuttling (Shrum et al., 2009). Interestingly, a recent report (Lee et al., 2019), identified the centrosomal accumulation of JLP (also known as SPAG9), a protein that is a critical regulator of nuclear translocation of P65. In our cells as well, we observed JLP accumulation at the centrosome from 15 mins after IL-1β stimulation (Fig. 6G). In contrast to the changes in the dynamics of dynein-dependent translocation of P65 (Fig. 1), we were unable to qualitatively see any differences between control and IFT88 depleted cells in JLP accumulation at the centrosome by 30 min. TTBK2 depletion similarly had no qualitative effect on JLP accumulation. As such, we would propose that it is most likely that IFT88, and potentially other ciliary proteins such as TTBK2, KIF3A and NPHP4, all exert their influence on the NFκB pathway at the IKK–IKB–P65 axis prior to or during trafficking of P65, upstream of the centrosome, and in doing so specifying the downstream transcriptional response to pro-inflammatory cues.

## DISCUSSION

We have previously shown that mutation of IFT88 regulates the cell response to IL-1β, and observed changes to IKK activity and IκB degradation shortly after cytokine exposure. This led us to propose that IFT88, most famously associated with primary cilia assembly and function, had a role in NFκB transduction, analogous to its roles in mechanotransduction (Wann et al., 2012) and particularly the regulation of Hedgehog (Hh) signalling, where the cilium offers a scaffold for tuning the activity of Hh transcription factors (Bangs and Anderson, 2017). The scope of influence this exerts over the spectrum of cell behaviours altered in inflammatory conditions remained unknown. We hypothesized the cilium may play a role in encoding inflammatory signalling, to facilitate the context-appropriate tuning of complex responses. We, and others, have observed changes to cilia structure and trafficking (Wann and Knight, 2012; Wann et al., 2013) in response to inflammatory cues, consistent with the concept of ciliary IFT proteins being recruited to inflammatory signal transduction. It was, prior to this study, unclear as to how important the ciliary axoneme was to this novel role for IFT88. In contrast to the early regulatory roles for IFT88, changes to the ciliary structure take hours, and are thus perhaps providing feedback to, or simply a later indicator of, earlier activity within the axonemal compartment. It remains unclear what role ciliary elongation has in inflammation. Changes in cilia length and assembly are associated with ciliopathies, for example cystic disease (Augereau et al., 2016), but also inflammatory disease sites, such as atherosclerosis (Dinsmore and Reiter, 2016). The data presented here imply that the role of ciliary proteins in inflammation may be more complex than canonical ciliary function as the cilium, per se, does not appear to be involved in early inflammatory signalling. We find that IFT88 disruption through mutation, depletion or deletion inhibits NFκB-dependent responses to inflammatory cytokines in ciliated and non-ciliated cells. Depletion of KIF3A, the kinesin responsible for anterograde IFT, including IFT88 trafficking, which normally phenocopies an IFT88 effect, has no inhibitory effect, but a trend towards an enhanced inflammatory response. This is in contrast to the positive regulatory role in the context of hippocampal neuron response to LPS (Baek et al., 2017), which may reflect context-dependent roles for KIF3A, particularly in cells types such as neurons where its non-ciliary roles are well-documented.

Similarly, depletion or overexpression of TTBK2, a kinase involved in the uncapping of the mature basal body enabling ciliary axoneme extension, also shows a trend towards the enhanced response to inflammatory cytokine. Further to this, overexpression of TTBK2 also mildly increased iNOS, indicative of a role for TTBK2 in the inflammatory response, but probably an indirect role. Conversely, depletion of the ciliary transition zone protein NPHP4, which is also cytokine-regulated, inhibits the response. Together with our finding that the ciliary protein IFT88 is also expressed at low levels in non-ciliated bone marrow-derived macrophages, our data support a role for multiple components of the ciliary/centrosomal/basal body machinery, independent of their roles in axonemal trafficking, in the regulation of response to inflammatory cytokine. We speculate accumulation of P65 at the basal body/centrosome is only indicative of late transit through this site as a result of cytoplasmic dynein trafficking demonstrated previously (Shrum et al., 2009), which has more recently been shown to be JLP dependent (Lee et al., 2019). This apparent centrosomal signal does not indicate where IFT88-mediated regulation of NFκB takes place. It is also important to highlight that we were unable to confirm localization of P65 with a mouse antibody to P65, due to non-specific binding, nor of a GFP-tagged P65. Centrosomal antibody staining for P65 changed with time and, for example, was not seen after nuclear translocation was complete beyond 30 min. Ciliary proteins, may contribute to an important regulatory nexus, upstream of transcription, as seen for Gli regulation in Hh signalling, albeit, in this context, not within the axoneme. An ambitious coupling of spatial proteomics, microscopy and transcriptional profiling will be required to explore this proposal further. IFT88 and other ciliary proteins may be involved in the carefully timed upstream transport of P65 towards the centrosomal/nuclear pore structure as described by Lee et al. (2019).

Many studies have shown changes to the cytoskeletal structure with perturbations of ciliary proteins such as IFT88. We have no evidence that the cytoskeleton is dramatically altered by IFT88 depletion in our experimental conditions, but changes to transport are possible. If so, it would seem, among pathways activated by cytokine, NFκB is most susceptible to these changes.

Studies of experimental inflammatory atherosclerosis, which is altered by conditional deletion of *Ift88* and thus removal of cilia (Dinsmore and Reiter, 2016), and the ciliopathy polycystic kidney disease (PKD) implicate cilia/ciliary proteins in inflammatory components of pathogenesis (Viau et al., 2018; Zimmerman et al., 2018). Macrophages have not been observed to assemble primary cilia, but macrophage activation and infiltration is altered in IFT88^ORPK^ mice (Zimmerman et al., 2018) and mice with a dysfunctional ciliary molecular complex (Viau et al., 2018). Thus, our findings may explain, in part, aberrant the inflammation seen in ciliopathies such as PKD where there is renewed focus on inflammation and the role of macrophages. Our studies used bone marrow-derived macrophages, which we find do express IFT88, in contrast to tissue-resident populations (Knoll et al., 1994); hence, this role of ciliary proteins may be cell type-specific and would require further investigation. IFT88 has been shown to be part of a complex with the Golgi-associated IFT protein IFT20, which promotes TCR clustering at the immune synapse in T cells (Finetti et al., 2009). A role for IFT88 in post-Golgi trafficking in inflammatory cell biology may well extend beyond the response to cytokine in non-ciliated immune cells. It remains unknown whether ciliary proteins play previously unheralded but complex, context-dependent roles in tissue inflammation and inflammatory disease.

A limited appreciation of how cascades, such as NFκB signalling, encode specificity to cellular responses downstream of inflammatory cues precludes successful therapeutic interventions in inflammation. There are a number of examples that indicate that cascade timing, often indicated by changes to NFκB shuttling, determine and, perhaps encode, transcriptional output (Mitra et al., 2017). IFT88 exerts some regulatory control over this, apparently affecting the timing of the key events in the IKK–IκB–P65 axis (Wann et al., 2014), and thus P65 shuttling rates (Fig. 1). Given the transport protein nature of IFT88 structure and the recent non-ciliary, cytoskeletal roles for IFTs (Bizet et al., 2015) and other components of the ciliome, including TTBK2 (Watanabe et al., 2015), we propose either a non-ciliary role for IFT88 in P65 transport dynamics [IFT proteins have already been shown to bind dynein (Delaval et al., 2011)], or the indirect disruption of this system through effects on the cytoskeleton, upstream of the centrosome. The compartmentalization of NFκB signalling components has been studied upstream and indicates a cytoplasmic dynein and/or centrosomal component (Vertii et al., 2016a,b,c), including an IKK signalsome (Kfoury et al., 2008), at the centrosome, where IκB has also been identified (Crépieux et al., 1997). It may be more difficult to image this collective when it is more diffuse in the cytosol during upstream signalling. Downstream of NFκB shuttling and NFκB transcript binding (Wann et al., 2014), one third of the transcriptional output (shown here by RNAseq) is affected. IFT88 depletion was most influential over a smaller subset of genes, an effect evenly distributed across all clusters irrespective of transcriptional dynamics. Despite this selectivity of effect, gene enrichment analysis implies that IFT88 and, on the basis of our RNAi interventions, potentially other ciliary proteins, is involved in biologically important pathways and processes in inflammation. The enrichments discovered within the temporal clusters, including on genes within the BBSome and ciliary body, basal body and centrosome, also included the type II dynein component WDR34, a TAK1 regulator (Gao et al., 2009). However, our previous data suggests the activity of the cytokine receptor and master kinase TAK1 is intact in IFT88 mutant cells (Wann et al., 2014). The RNA-seq dataset can serve as a platform for further work substantiating our interpretation that IFT88 elicits response specificity through regulatory bias of NFκB over other signalling pathways. We speculate this is due to an NFκB reliance on active, dynein-dependent cytoskeletal trafficking.

In summary, we demonstrate that disruption of ciliary proteins alters the cellular response to inflammatory cues in both ciliated and non-ciliated cells. A specific subset of the cell response, one third of the response to IL-1, is impaired by disruption of IFT88. Responses are enhanced by KIF3A and TTBK2 targeting. Despite axonemal elongation and the cytokine-mediated induction of a subset of ciliary genes in response to inflammatory cues, we suggest that IFT88-mediated regulation of the inflammatory response is not indicative of a ciliary axonemal role in early inflammatory signalling. Indeed depletion of IFT88 in macrophages, which do not assemble primary cilia, reduces the response to pro-inflammatory cues. Given the potential universal effect of IFT88 in the context of cytokine response, combined with the selectivity of effect within inflammatory targets, we propose peripherally cytosolic, ‘ciliary’ proteins may be novel components in the encoding of inflammatory signalling, and therefore are an exciting set of proteins to explore in inflammatory signalling and approaches aiming to restore physiological inflammatory signalling in disease.

## MATERIALS AND METHODS

### Cell culture

A mouse chondrocyte cell line was used as previously described (Wann et al., 2012), and was recently authenticated and tested for mycoplasma contamination, in a base medium (all Sigma-Aldrich) of low-glucose DMEM (D5921), 10% (v/v) fetal calf serum (F7524), 2.5 mM L-glutamine (G7513), 88 U ml^−1^ penicillin and 90 µg ml^−1^ streptomycin (P4333). Experiments were conducted with primary cells [released from conditional immortalization induced with 33°C culture in the presence of 10 ng ml^−1^ mouse recombinant interferon-γ (R&D systems) by washing and culturing cells at 37°C without INF-γ]. Cells were seeded in complete mouse primary culture medium for experiments. Articular cartilage was isolated from the metacarpophalangeal joint and incubated overnight at 37°C in 1 mg/ml collagenase (Sigma-Aldrich, UK), in porcine culture medium (as cell line medium plus 16 mM HEPES). The cells released were strained and centrifuged, followed by removal of the supernatant and resuspension of the cell pellet in fresh porcine culture medium. Cells were seeded onto sterile 1.9 cm^2^ glass coverslips at 8×10^4^ cells cm^−2^. The culture medium was changed on the 6th and 13th day post seeding and cells were used for experiments on the 15th day, once they had attached to the glass culture surface and quiescence was reached.

For macrophage studies, bone marrow was collected from 7-week-old IFT88*^fl/fl^/*ROSA26ERT^2^ mice (B6;129-*Gt(ROSA)26Sortm1(cre/ERT)Nat*/J; stock no. 004847, The Jackson Laboratory), suspended in freezing medium (10% DMSO in FBS) and stored for later use. All animal experiments were performed according to approved guidelines. Murine bone marrow-derived macrophages (BMDMs) were differentiated from total bone marrow cells cultured for 7 days in BMDM medium [RPM1 1640 (Thermo Fisher, cat. number 21870076) supplemented with 10% heat inactivated FBS (Gibco certified low endotoxin, cat. number 1600044), 1× penicillin-streptomycin-glutamine (100× stock, Thermo Fisher cat. number 10378016), 25 mM HEPES (1 M stock, Thermo Fisher cat. number 15630056), 1 mM Na-pyruvate (100× stock, Thermo Fisher cat. number 11360070) and 50 ng/ml recombinant human macrophage colony-stimulating factor (Immunotools, endotoxin free, cat. number 11343117)]. Cells were fed on day 5 of differentiation with fresh BMDM medium and at that time treated with 500 nM 4-hydroxytamoxifen (Sigma Aldrich, T176) or vehicle (ethanol) for 48 h. On day 7, cells were lifted with cold PBS and seeded at 1×10^6^ cells ml^−1^ in 12-well plates in a total volume of 1 ml, in 4-hydroxytamoxifen-free BMDM medium. Cells were allowed to adhere for 5–6 h and then stimulated with 100 ng ml^−1^ ultrapure *Escherichia coli* K12 LPS (Invitrogen, cat number tlrl-peklps), 10 ng ml^−1^ endotoxin-free recombinant murine TNF (Biolegend, career free, cat number 575204) or 10 ng ml^−1^ endotoxin-free recombinant human IL-1β (PeproTech, cat number 200-01B). Deletion of IFT88 and induction of iNOS mRNA was analysed by qPCR.

### Cytokine inductions in non-macrophage cells

IL-1β (Peprotech) and TNF (Invitrogen) were diluted in PBS containing 0.1% BSA before dilution in media depending on experiment and used at concentrations as indicated in results or experimental sections in the Materials and Methods.

### Antibodies

Primary antibodies used in this study are as follows: β-actin (Novus Biologicals, 8H10D10 1:5000), acetylated α-tubulin (clone 6-11B-1, Sigma-Aldrich T7541 1:2000), arl13b (Proteintech, 17711-1-AP 1:1000), IFT88 (Proteintech, 13967-1-AP 1:500), IFT88 (Fig. 5B) (Santa Cruz Biotchnology, sc-84318 1:250), KIF3a (Abcam, ab11259 1:500), COX2 (Cell Signaling Technology, D5H5/12282 1:500), iNOS (Cell Signaling Technology D6B6S/13120 1:500), NFκB p65 (Abcam, ab7970 1:500), IKB (Abcam, ab32518 1:250), phospho-JNK1/2 (Thermo Fisher Scientific, D12H7L17) 1:500, phospho-ERK1/2 (Cell Signaling Technology, 9101 1:500). JLP/SPAG9 (D72F4) rabbit monoclonal antibody (Proteintech 5519 1:50), and γ-tubulin clone GTU-88 mouse monoclonal (Sigma, T6657 1:1000). Alexa Fluor-conjugated secondary antibodies (Invitrogen) and DAPI (1:5000, counterstain, also Invitrogen) were used for immunofluorescence studies.

### siRNA knockdown

50–60% confluent murine chondrocytes were incubated in murine primary medium. Cells were transfected with 10 nM of siRNA (4× smart pools) for IFT88 [mouse Ift88 (Dharmacon, L-050417-00-0005)], KIF3A [mouse Kif3A (Dharmacon, L-042111-01-0005)], TTBK2 [mouse Ttbk2 (Dharmacon, L-047640-00-0005)], NPHP4 [mouse Nphp4 (Dharmacon, L-055006-01-0005) or DyneinC2H1 [mouse Dync2h1 (Dharmacon, M-061741-01-0005)] and a non-targeting control siRNA [non-targeting pool (Dharmacon, D-001810-10-05)] using RNAiMAX (Invitrogen), made up in optiMEM and cultured for 48 h. TTBK2 overexpression was conducted as per siRNA experiments, using mouse cDNA clone MR217963 (accession no. NM080788) TTBK2 Myc-DDK tagged transcript variant 1 through a pCMV6-entry vector purchased from Origene.

### RNA extraction and qPCR

Cells were cultured as described above then lysed for RNA collection using an RNAeasy mini kit (Qiagen). RNA was re-suspended in RNase-free water and yields and indicative purity checked using Nanodrop (Thermo Fisher Scientific). For WT versus IFT88^ORPK^ comparisons, 1 µg of RNA was used to synthesize cDNA, and reverse transcription was performed using the ABI High Capacity Kit (Thermo Fisher Scientific) following the manufacturer's instructions. Real-time RT-PCR was performed using the TaqMan (FAM dye) Universal Master Mix II (Thermo Fisher Scientific). Each reaction consisted of 0.5 µl cDNA template, 5 µl master mix, 0.5 µl primers, and 4 µl nuclease-free water. For WT versus IFT88^siRNA^ and RNA-seq validation, RNA integrity was assessed using an Agilent 2100 Bioanalyzer and the RNA Nano 6000 assay (Agilent Technologies) following the manufacturer's instructions. 500 ng RNA was used to synthesize cDNA. Reverse transcription was performed using the High Capacity cDNA Reverse Transcription Kit without RNase inhibitor (Thermo Fisher Scientific) following the manufacturer's instructions. cDNA from chondrocyte experiments was diluted 1:2.5 in RNase-free water before use in real-time qPCR. For macrophage qPCR in initial 4-hydroxytamoxifen testing, 500 ng RNA was used to synthesize cDNA. For experimental conditions, 1 µg RNA was used to synthesize cDNA. Reverse transcription was performed using the High Capacity cDNA Reverse Transcription Kit without RNase inhibitor (Thermo Fisher Scientific) following the manufacturer's instructions. cDNA from macrophage experiments was diluted 1:2 in RNase-free water before qPCR. All qPCR was performed using the TaqMan Fast Universal PCR Master Mix (Thermo Fisher Scientific, UK). Each reaction comprised 2.4 µl cDNA template, 3 µl Master Mix, 0.3 µl primer and 0.3 µl nuclease-free water. Samples were loaded in a 384-well plate and thermocycling was performed on a ViiA7 Real-Time PCR System (Thermo Fisher Scientific) using the following protocol: hold 2 min at 50°C; hold 10 min at 95°C; 40 cycles: 15 s at 95°C, 1 min at 60°C; hold at 4°C. Data were captured and primary analysis performed using Expression Suite Software v 1.1 (Applied Biosystems, Warrington, UK) by the ΔΔCT method using 18S as a normalizing gene, identified as most stable of three potential references checked (18S, B2 M and GAPDH). TaqMan assays, as below, were purchased from Thermofisher Scientific, UK. Sequences of primers are available upon request.

### Western blotting

Cells were washed on ice quickly in 500 µl of ice cold PBS before 300 µl of ice cold lysis buffer [150 mM sodium chloride, 1% Triton-X 100, 50 mM Tris pH 8, a cocktail of protease inhibitors (Roche)] was added to the cells for 5 min. Cells were removed from the culture surface using a cell scraper before being left for a further 5 min and homogenized through a 21 G needle. Samples were spun at 8000 ***g*** for 10 min at 4°C before the supernatant (cytoplasmic) fraction was transferred to a fresh tube, frozen and stored at −20°C for later use. Proteins were resolved on Bis-Tris gels (Thermo Fisher Scientific, UK) and transferred using the BioRad transblotting system. ImageJ was used to quantify immunoreactive bands labelled using LI-COR near-infrared secondary antibodies, multiplexing target protein with reference.

### Immunofluorescence

Coverslip cultures were fixed using 4% paraformaldehyde at 37°C for 7 min, followed by permeabilization with 0.5% Triton X-100 and blocking with 5% goat serum. Coverslips were incubated overnight at 4°C with primary antibodies depending on protein of interest [acetylated a-tubulin (1:2000), Arl13b (1:1000), NFκB P65 (1:500)]. Coverslips were washed, incubated for 45 min in the dark at 25°C with Alexa Fluor 488-conjugated anti-mouse-IgG and Alexa Fluor 555-conjugated anti-rabbit-IgG (Invitrogen), before DAPI (1:5000) counterstaining (Invitrogen) and mounting in Prolong gold mountant.

### Imaging

High resolution microscopy was conducted using an Olympus FluoView FV1000 confocal microscope with an oil immersion 63× objective to produce confocal serial sections (pixel size 150 nm), for maximum intensity *z*-stack reconstruction of monolayer fields. Lower resolution images were taken using a Zeiss Axioscope. TIRFM was conducted using an Olympus TIRF IX83, imaged with an UAPON 150×/NA 1.45 oil objective.

### Cilia length

WT cells or primary porcine chondrocytes were cultured to confluence on 1.9 cm^2^ glass coverslips before being treated with 10 ng/ml IL-1β or TNF for 24 h. Cells were fixed and prepared as described above using primary antibodies against acetylated α-tubulin (1:2000) and Arl13b (1:1000). Confocal microscopy was used to acquire images, and projected cilia length was measured from a maximum intensity *z*-stack reconstruction, using a medial line defined along the centre of the cilium using ImageJ software.

### NFκB P65 nuclear intensity and cytoplasmic localization

WT and ORPK chondrocytes were cultured to confluence on 1.9 cm^2^ glass coverslips before being treated with 10 ng/ml IL-1β or TNF for 10, 20 and 30 min time points. Cells were fixed and prepared as described above using primary antibodies against acetylated α-tubulin (1:2000) and NFκB P65 (1:500). Epifluorescence wide-field microscopy was used to acquire wide-field images of cell preparations. Using ImageJ, the DAPI-labelled nuclei were defined as areas of interest and the P65 signal intensity was measured. Cells were identified as ciliated or non-ciliated based on acetylated α-tubulin ciliary labelling.

### RNA-seq experiment

WT cells were cultured in 6-well culture plates, directly into primary cell medium at a cell density of 15,000 cells cm^−2^ in 2 ml total volume. Cells were then cultured to 50–60% confluence and transfected with an IFT88 siRNA as described above. Cells were then treated with 10 ng/ml IL-1β for 1 h 2 h, 4 h, 8 h and 24 h with an untreated medium only, as a control, before being lysed in ice-cold RLT lysis buffer (Qiagen) and stored at −20°C, ready for RNA extraction, as described above. All RNA RIN values were determined to be >9. PolyA-selected sequencing libraries were prepared using the TruSeq protocol (Illumina). Libraries were subject to 75 bp paired-end sequencing (Illumina HiSeq 4000) to an average depth of 28.5 million read pairs per sample.

### RNA-seq data analysis

Sequence reads were aligned to the mouse genome with Hisat2 (version 2.1.0) (Kim et al., 2015) using a ‘genome_trans’ index built from the GRCm38 release of the mouse genome and Ensembl version 91 annotations (two-pass strategy to discover novel splice sites; with parameters: --dta and --score-min L,0.0,-0.2). Data quality was assessed using the pipeline_readqc.py script (https://github.com/cgat-developers/cgat-flow/). The average alignment rate was 97.25% (as assessed with picard tools v2.10.9, https://github.com/broadinstitute/picard). Mapped reads were counted using featureCounts (Subread version 1.6.3; Ensembl version 91 annotations; with default parameters) (Liao et al., 2014). Salmon v0.9.1 was used to calculate transcripts per kilobase million (TPM) values (Patro et al., 2017) using a quasi-index (built with Ensembl version 91 annotations and k=31) and gc bias correction (parameter “--gcBias”). Time-course analysis was performed using ImpulseDE2 (v 1.6.0) (https://doi.org/10.1101/113548). Genes that showed significant changes [Benjamini–Hochberg (BH) adjusted *P*<0.05; |fold change|>1.5] following IL-1β treatment in the non-targeting (NT) samples (case-only analysis) were subject to k-means clustering analysis (Fig. 3A). Genes whose response to IL-1β was modulated by IFT88^siRNA^ were then identified by intersecting the results of case-control analysis (BH adjusted *P*<0.05; |fold change|>1.5) with the NT case-only results (Fig. 3C). Over-represented genesets (one-sided Fishers exact tests; BH adjusted *P*<0.05) were identified with gsfisher (https://github.com/sansomlab/gsfisher). Pertime point differential expression analysis of IFT88 treated versus control samples was performed using DESeq2 (v1.22.0) (Love et al., 2014).

### Protease activity

Cells were co-cultured with aggrecan, before probing for extracellular (medium) neoepitopes, as described previously (Ismail et al., 2015). In brief, confluent cells, as per other experiments, were incubated with bovine aggrecan (50 µg/ml) with or without 10 ng/ml IL-1β. Medium was collected after 24 h and before analysis deglycosylated overnight, concentrated by acetone precipitation and denatured before probing for immunoreactive bands signifying fragments generated by protease cleavage at the Glu^1819^-Ala^1820^ bond in aggrecan in western blots using a polyclonal rabbit antibody directed at N-terminal AGEG at 1:500 (kind gift from Hideaki Nagase, University of Oxford, UK).

### Griess assay

NO was measured in the culture medium following 24 h IL-1β treatment using a Greiss assay kit (Insight Biotechnology, cat number 30100) according to the manufacturer's instructions, and against a sodium nitrite standard measured at 544 nm.

### Data presentation and statistical analysis

Graphs were drawn in GraphPad Prism (GraphPad Software, La Jolla, CA). All data manipulations and statistical analyses were performed in Graph Pad Prism. For the purposes of statistical significance *P<0.05, **P<0.01, ***P<0.001, ****P<0.0001. Specifics of graphical representation, statistical tests and *n* numbers are defined in the respective figure legends.

## Supplementary Material

Supplementary information

Reviewer comments
