# Supplementary material for: Ciliary proteins specify the cell inflammatory response by tuning NFκB signalling, independently of primary cilia
Source: J Cell Sci. 2020 Jul 8;133(13):jcs239871. doi: 10.1242/jcs.239871 (PMC7358134; doi:10.1242/jcs.239871)
Supplement: Supplementary information [file joces-133-239871-s1.pdf]

Supplementary materials

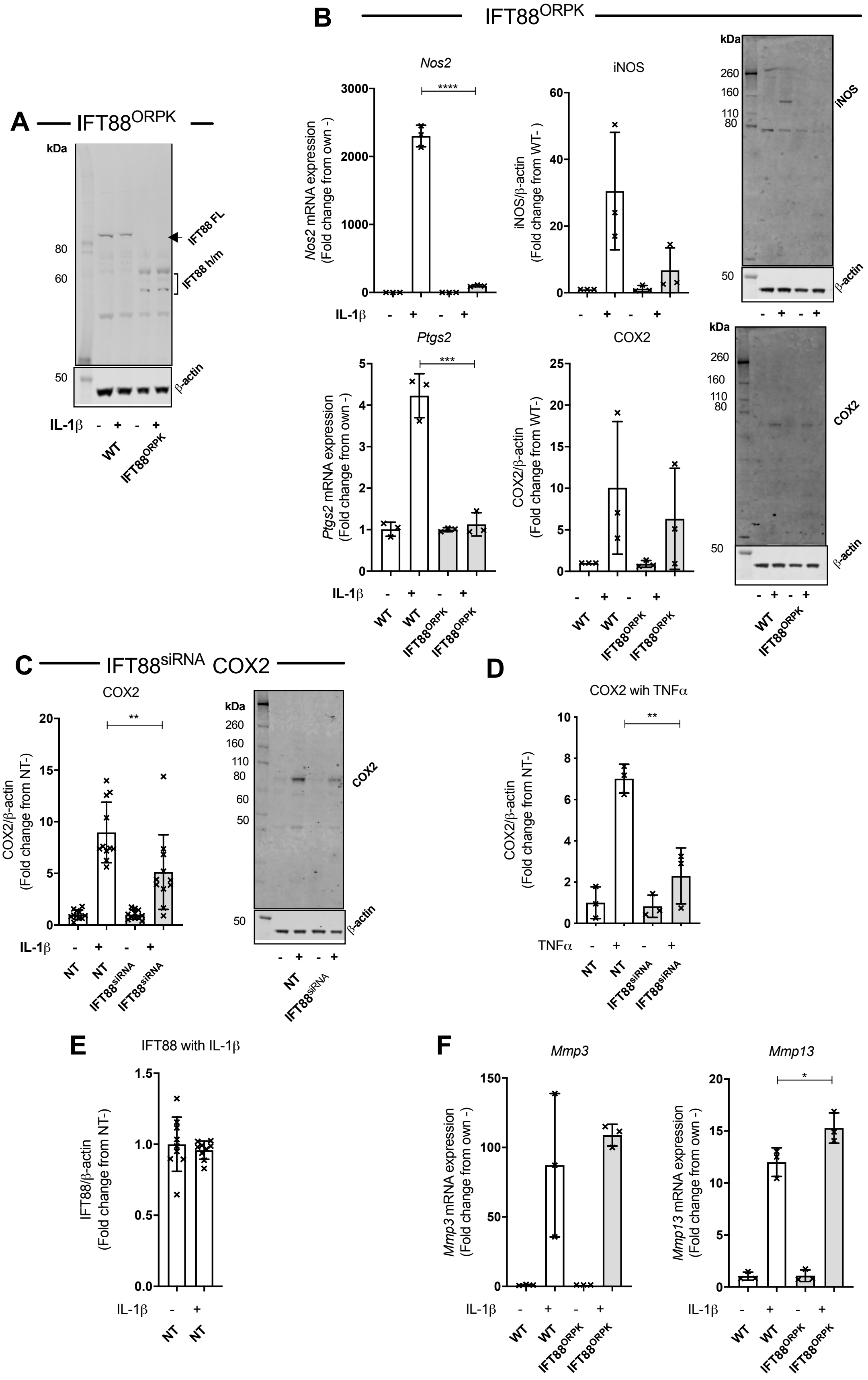

Journal of Cell Science • Supplementary information

Fig. S1 Disruption of IFT88 alters cell response to IL-1β and TNFα

A Western blot analysis of WT and IFT88^{ORPK} cell lysates, from cells cultured ± 10ng/ml IL-1β for 24hr, probing for IFT88 and β-actin as a loading control. **B** *Ptgs2* mRNA expression (top, left) and COX2 protein expression (top, right) in WT and IFT88^{ORPK} cells cultured ± 10ng/ml IL-1β for 24hr. *Ptgs2* presented as a fold change from the mean *Ptgs2 expression* of the -IL-1β condition (*n*=3, ***P<0.0001, Student's *t*-test. *t*=8.948 df=4). COX2 levels presented as a fold change from mean COX2/β-actin levels of the –IL-1β WT condition (*n*=3, ns *P*=0.0949, Student's *t*-test. *t*=2.179, df=4). *Nos2* mRNA (bottom, left) and iNOS protein expression (bottom, right) in WT and IFT88^{ORPK} cells cultured ± 10ng/ml IL-1β for 24hr. *Nos2* presented as a fold change from the mean *Nos2* expression of the -IL-1β condition (*n*=3, ****P<0.0001, Student's *t*-test. *t*=23.98, df=4). iNOS levels presented as a fold change from the mean iNOS/β-actin in the –IL-1β WT condition (*n*=3, ns *P*=0.5548, Student's *t*-test. *t*=0.6437, df=4). All graphs presented as the mean±SD. **C** COX2 protein expression in NT and IFT88^{siRNA} cells cultured ± 10ng/ml IL-1β for 24hr. Protein levels were quantified and the data presented as a fold change from mean expression / β-actin levels of the –IL-1β NT condition, *n*=11, **P<0.001, Mann-Whitney test. U=18). All graphs presented as the mean±SD. **D** COX2 protein expression in NT and IFT88^{siRNA} cells cultured ± 10ng/ml TNFα, for 24hr. COX2 levels presented as a fold change from mean COX2/β-actin levels of the –IL-1β NT condition (*n*=3, **P=0.0059, Student's *t*-test. *t*=5.357 df=4). **E** IFT88 protein expression in NT cells cultured ± 10ng/ml IL-1β for 24hr. IFT88 levels were quantified and the data presented as a fold change from the mean IFT88/β-actin levels of the –IL-1β NT condition (*n*=9, ns *P*=0.5445, Student's *t*-test. t=0.6192 df=16). All graphs presented as mean±SD. **F** *Mmp3* and **b** *Mmp13* mRNA expression in WT and IFT88^{ORPK} cells cultured ± 10ng/ml IL-1β for 24hr, p sented as a fold change from the mean of the WT - IL-1β condition (*n*=3, **a** ns, **b** *P=0.464, Student's *t*-test. *t*=2.849 df=4).

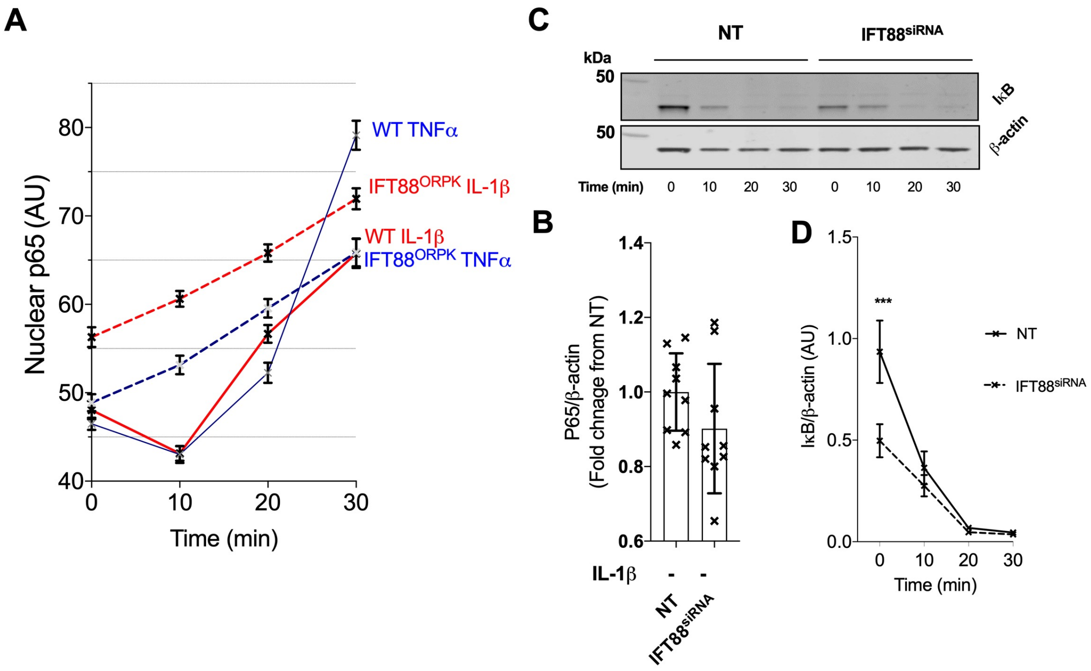

Figure. S2 Effects of IFT88 disruption on NFκB signalling dynamics.

A P65 translocation curves for WT and ORPK conditions without normalisation to T=0hr. Quantification of IF nuclear p65 intensity in WT (solid line) and IFT88^{ORPK} (dashed line) cells. 30min time course of 10ng/ml IL-1β (red) or TNFα (blue), with 10, 20 and 30min cytokine time points and an unstimulated media only 0hr time point (n = 140 to 200 nuclei per condition, mean±SEM). **B** Western blot analysis of IκB levels in NT and IFT88^{siRNA} cells cultured ± 10ng/ml IL-1β for 0, 10, 20 and 30min presented as IKB/β-actin, β-actin as a loading control. (n=9, p=0.164) **C** P65 protein expression in NT and IFT88^{siRNA} cells, presented as P65/β-actin levels of the condition (n=3, ns, P>0.05. Students t-test, mean±SD). **D** IKB protein expression levels in NT and IFT88^{siRNA} cells cultured ± 10ng/ml IL-1β for 0min, 10min, 20min and 30min (NT and IFT88 n=8 for all time points). Proteins levels presented normalised to the levels of the loading control β-actin (***P=0.0002, two-way ANOVA, mean±SEM).

Journal of Cell Science • Supplementary information

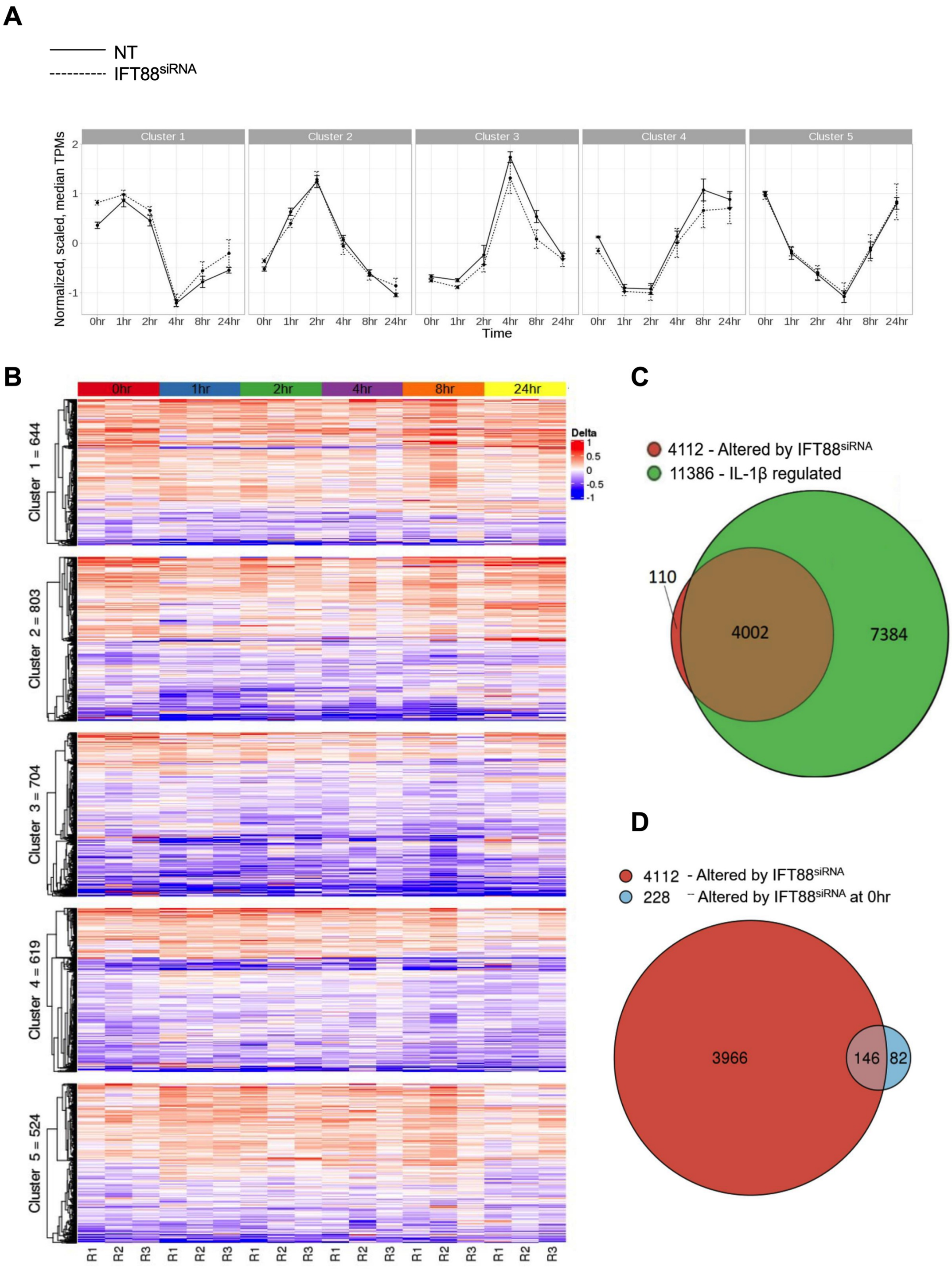

Journal of Cell Science • Supplementary information

Fig S3 Effect of IFT88 depletion on genome-wide (RNAseq) transcriptional response to IL-1β

A Global temporal profiles over the 24hr time course of the 5 clusters of IL-1β regulated genes (solid line) and how IFT88^{siRNA} globally alters these profiles (dashed line; graph presented as mean±SD).**B** Heat map for clusters 1 to 5, representing the relative gene expression as either upregulated (red) or downregulated (blue) in the IFT88^{siRNA} condition as compared to NT condition over the 24hr time course. **C** Venn diagram of gene expression across the 24hr IL-1β time course, with the number of genes regulated by cytokine in green and the number of genes altered over the time-course by IFT88 siRNA depletion in red (BH adjusted $P<0.05$; fold change > 1.5; ImpulseDE2 case-control analysis). **D** Venn diagram showing the number of genes altered across the time course by IFT88 siRNA depletion, as in c and thus in red, and the number of genes altered at baseline (0hr) by IFT88 siRNA depletion in blue (BH adjusted $P<0.05$; fold change > 1.5; ImpulseDE2 case-control analysis).

Journal of Cell Science • Supplementary information

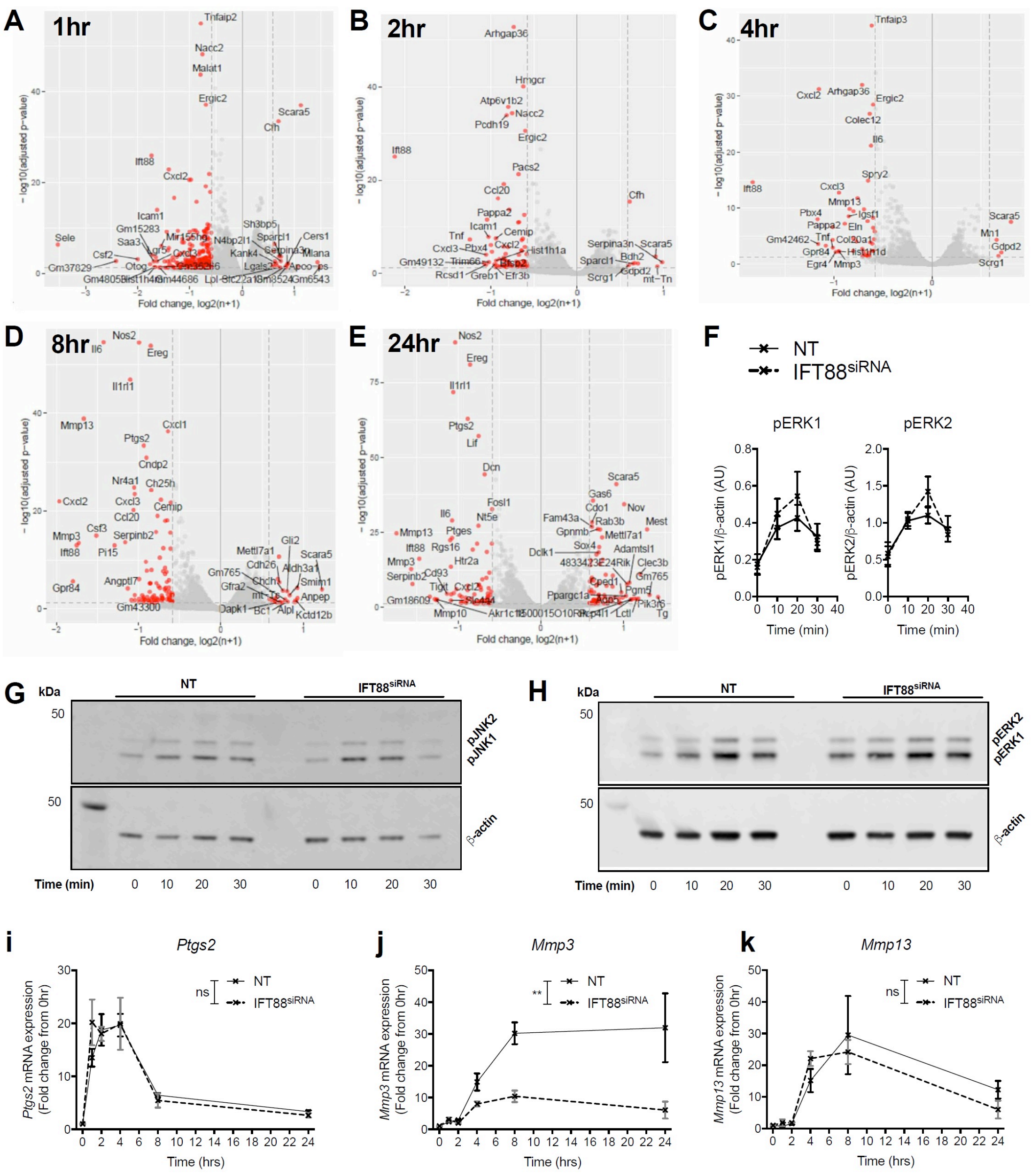

Fig. S4 Effect of IFT88 depletion on genome-wide (RNAseq) transcriptional response to IL-1β (continued).

A-E Volcano plots for each time point, representing the differential expression of genes in the IFT88^{siRNA} condition with respect to the NT control, for each time point. Red points represent significantly different (*FDR*<0.05) genes up (to the right) or down (to the left) in the IFT88^{siRNA} condition as compared to the NT control (BH-adjusted *P*<0.05; fold change > 1.5; DESeq2 analysis). The magnitude of the fold change is on the x-axis with the magnitude of significance on the y-axis. **F**

Journal of Cell Science • Supplementary information

pERK1 (left) and pERK2 (right) protein expression levels in NT and IFT88^{siRNA} cells cultured ± 10ng/ml IL-1β for 0min (NT *n*=7 and IFT88 ^{siRNA} *n*=6), 10min (NT *n*=7 and IFT88 ^{siRNA} *n*=6), 20min (NT *n*=4 and IFT88 ^{siRNA} *n*=3) and 30min (NT and IFT88 *n*=6). Graphs presented as mean±SEM (ns, two-way ANOVA). **G, H** Western blot analysis of pJNK (**G**) and pERK (**H**) in NT and IFT88^{siRNA} cells cultured with 10ng/ml IL-1β for 0, 10, 20 and 30min, β-actin as a loading control. **I** *Ptgs2,* **J** *Mmp3* and **K** *Mmp13* mRNA expression in NT (solid line) and IFT88^{siRNA} (dashed line) cells cultured with 10ng/ml IL-1β for a time course of 1, 2, 4, 8 and 24hr, presented as a fold change from their respective 0hr unstimulated controls (*n*=3 for each condition at each time point). All graphs presented as mean±SD with two-way ANOVA analysis performed (ns P>0.05, **P<0.01).

Journal of Cell Science • Supplementary information

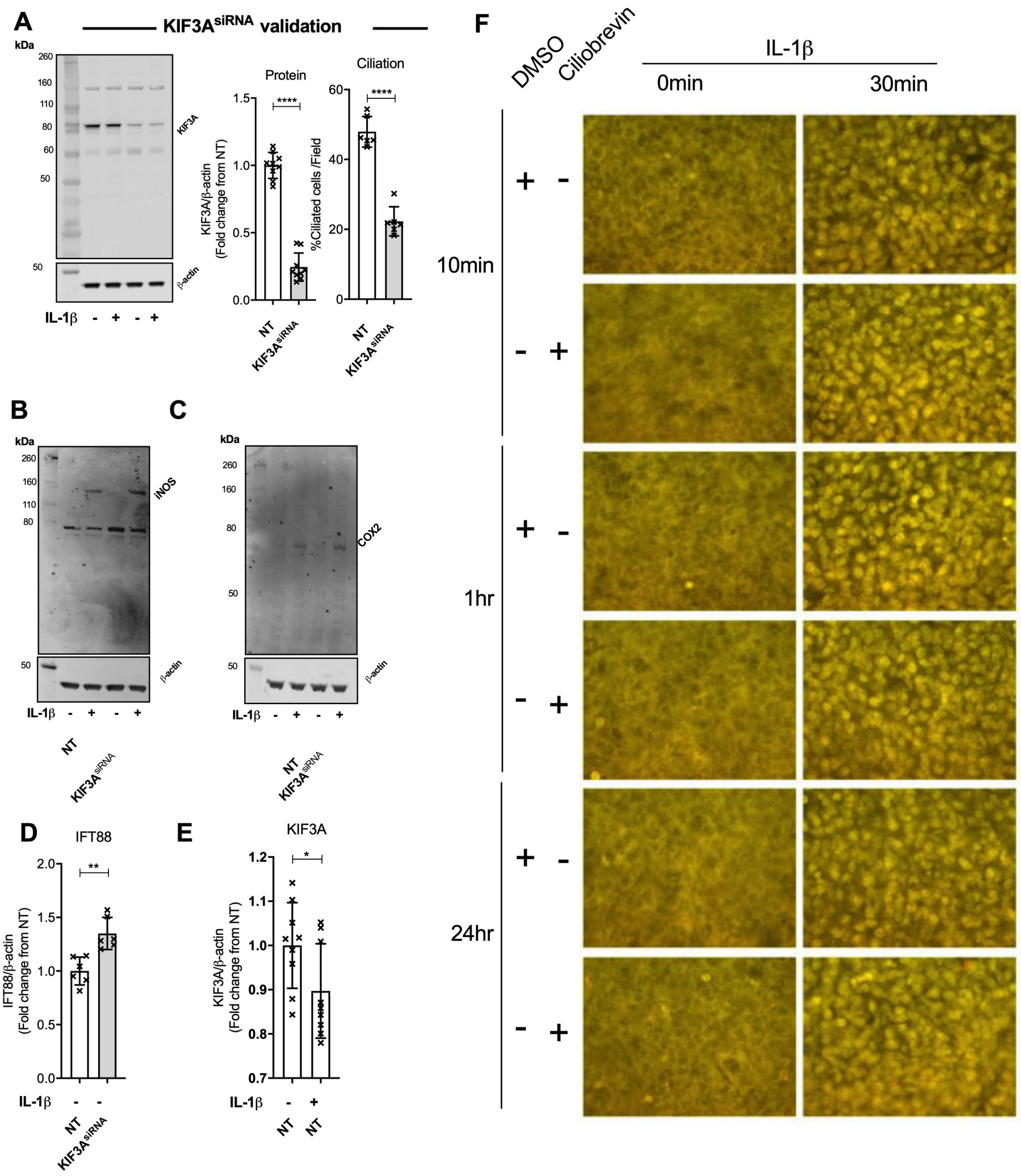

Fig. S5. Effects of KIF3A depletion

A Western blot analysis probing for KIF3A (left) in NT and KIF3A^{siRNA} cell lysates, from cells cultured ± 10ng/ml IL-1β for 24hr. KIF3A quantified (middle) and data presented as a fold change from mean KIF3A/β-actin of the –IL-1β NT condition (n=9, ****P<0.0001, Student's t-test. t=15.89 df=16). Effect of KIF3A^{siRNA} on cilia (right), shown as percentage ciliated cells in NT and KIF3A^{siRNA} cell cultures (n=6 fields from two repeats, ****P<0.0001, Fisher's exact test). **B/C** Representative western blots showing iNOS **(B)** and COX2 **(C)** protein expression in NT and

Journal of Cell Science • Supplementary information

KIF3A^{siRNA} cells cultured ± 10ng/ml IL-1β for 24hrs. **D** IFT88 protein expression in NT and KIF3A^{siRNA}. IFT88 levels presented as a fold change from mean IFT88/β-actin levels of the NT condition (n=6 for each condition, ***P*=0.0015, Students *t*-test. *t*=4.326 df=10). **E** KIF3A protein expression in NT cells cultured ± 10ng/ml IL-1β for 24hr. KIF3A levels presented as a fold change from mean KIF3A/β-actin levels of the –IL-1β NT condition (*n*=9 for each condition, **P*=0.0478, Students *t*-test. *t*=2.144 df=16). **F** IF microcopy of p65 localisation in WT chondrocytes, treated with 30μM Ciliobrevin D or the vehicle control DMSO for 10min, 1hr or 24hr before ± 10ng/ml IL-1β for 30min. Scale bar 50μm.

Journal of Cell Science • Supplementary information

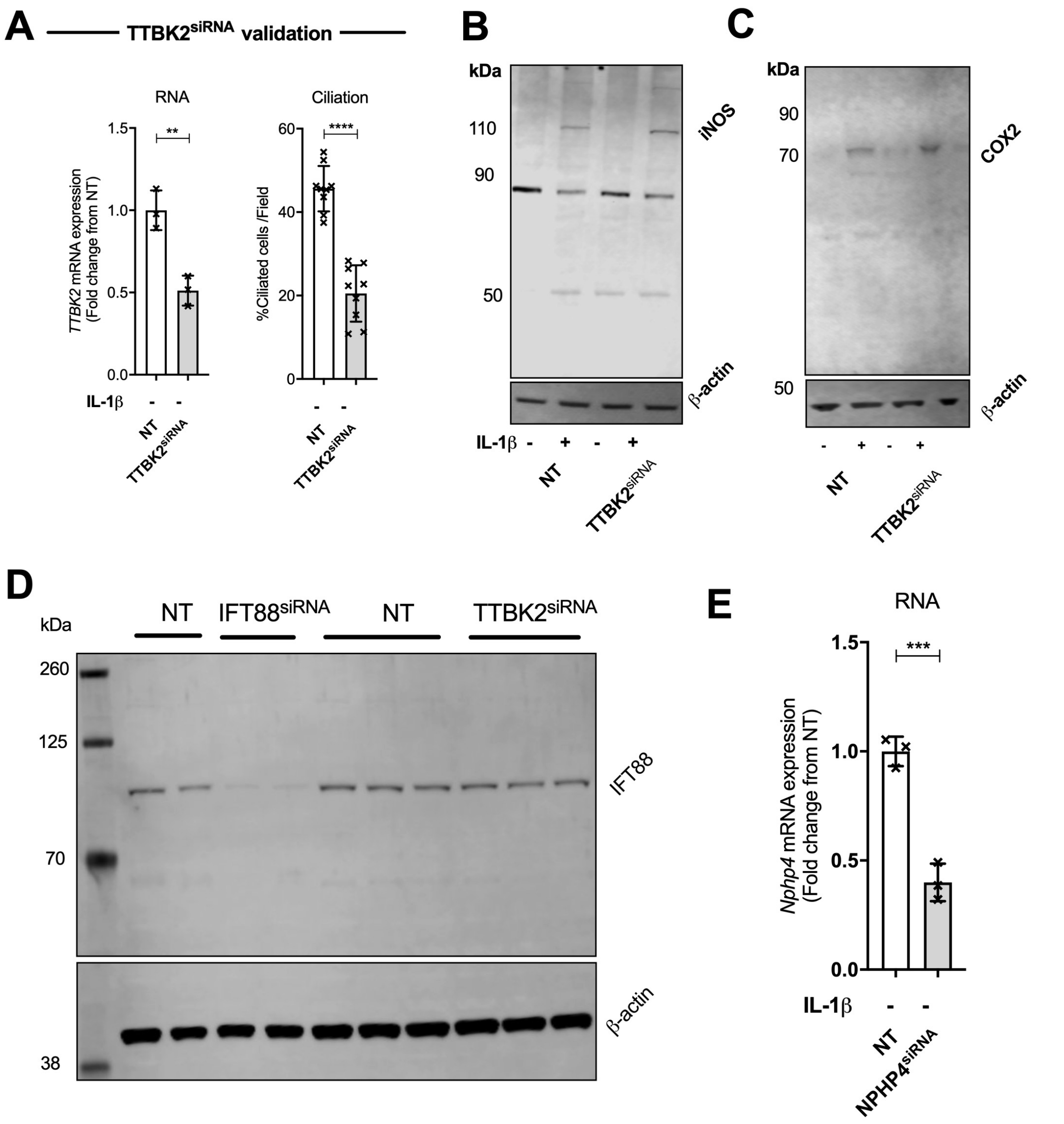

Fig S6 TTBK2/NPHP4 depletion

A TTBK2 mRNA expression in NT and TTBK2^{siRNA} cells (left). Data presented as a fold change from mean of NT condition (*n*=3, ***P*=0.005, Student's *t*-test. t=5.598 df=4). Effect of TTBK2^{siRNA} depletion on cilia (right), shown as percentage ciliated cells in NT and TTBK2^{siRNA} cell cultures (*n*=9 fields from three repeats, *****P*<0.0001, Fisher's exact test). **B/C** Representative western blots showing iNOS **(B)** and COX2 **(C)** protein expression in NT and TTBK2^{siRNA} cells cultured ± 10ng/ml IL-1β for 24hrs. **D** Western blot analysis of IFT88 with siRNA depletion of TTBK2, β-actin as a loading control. **E** *Nphp4* mRNA expression in NT and NPHP4^{siRNA} cells, data presented as a fold change from mean of the NT condition (*n*=3, ****P*=0.0007, Student's *t*-test. *t*=9.55 df=4).

Journal of Cell Science • Supplementary information
