# Supplementary material for: Ciliary proteins specify the cell inflammatory response by tuning NFκB signalling, independently of primary cilia
Source: J Cell Sci. 2020 Jul 8;133(13):jcs239871. doi: 10.1242/jcs.239871 (PMC7358134; doi:10.1242/jcs.239871)
Supplement: Reviewer comments [file joces-133-239871_review_history.pdf]

Journal of Cell Science

Ciliary proteins specify the cell inflammatory response by tuning NFκB signalling, independently of primary cilia.

Megan Mc Fie, Lada Koneva, Isabella Collins, Clarissa R. Coveney, Aisling M. Clube, Anastasios Chanalaris, Tonia L. Vincent, Jelena S. Bezbradica, Stephen N. Sansom and Angus K. T. Wann
DOI: 10.1242/jcs.239871

Editor: David Stephens

Review timeline

Original submission:	2 October 2019
Editorial decision:	25 November 2019
First revision received:	20 February 2020
Editorial decision:	24 March 2020
Second revision received:	16 April 2020
Editorial decision:	14 May 2020
Third revision received:	19 May 2020
Accepted:	21 May 2020

Original submission

First decision letter

MS ID#: JOCES/2019/239871

MS TITLE: Ciliary proteins tune NFκB signalling independently of primary cilia to specify the cellular response to inflammatory cytokine

AUTHORS: Megan Mc Fie, Lada Koneva, Isabella Collins, Clarissa R Coveney, Aisling M Clube, Anastasios Chanalaris, Tonia L Vincent, Jelena S Bezbradica, Stephen N Sansom, and Angus KT Wann
ARTICLE TYPE: Research Article

We have now reached a decision on the above manuscript.

To see the reviewers' reports and a copy of this decision letter, please go to: https://submit-jcs.biologists.org and click on the 'Manuscripts with Decisions' queue in the Author Area. (Corresponding author only has access to reviews.)

As you will see, the reviewers raise a number of substantial criticisms that prevent me from accepting the paper at this stage. They suggest, however, that a revised version might prove acceptable, if you can address their concerns. If you think that you can deal satisfactorily with the criticisms on revision, I would be pleased to see a revised manuscript. We would then return it to the reviewers.

You will see that one reviewer raises some significant concerns. I do not consider that genome editing is necessary to support your story at this point. However, I do agree with them that further controls are needed, in particular resolution of the IFT88 depletion in macrophages and further validation of key outcomes using "rescue" experiments of key RNAi targets.

Please ensure that you clearly highlight all changes made in the revised manuscript. Please avoid using 'Tracked changes' in Word files as these are lost in PDF conversion.

© 2020. Published by The Company of Biologists under the terms of the Creative Commons Attribution License (https://creativecommons.org/licenses/by/4.0/).

I should be grateful if you would also provide a point-by-point response detailing how you have dealt with the points raised by the reviewers in the 'Response to Reviewers' box. Please attend to all of the reviewers' comments. If you do not agree with any of their criticisms or suggestions please explain clearly why this is so.

Reviewer 1

Advance summary and potential significance to field

The manuscript by McFie et al., builds upon previous research indicating that cilia proteins participate in cytokine signalling, namely the response to IL-1B.
IN this manuscript the authors perform RNA-seq to identify differential expression of IL-1B responsive genes when IFT88 is transiently knocked down by siRNA.

My major concern with this manuscript is that it adds little to the current knowledge of how cilia proteins regulate the IL-1B response. There is no real mechanistic insight provided. In fact, experiments where knockdown of other cilia-associated genes such as Kif3a and TTBK2 give the opposite effect to IFT88 depletion, somewhat confuse the field.

The authors speculate that IL-1B signalling is operating at the basal body, but provide no definitive evidence of this.

On the whole, the manuscript is well written and the for the most part appropriate controls used, however, in its current state does not provide a significant advancement to the field and being mostly descriptive, adds no mechanistic insight.

Comments for the author

Some major controls are lacking in the siRNA experiments. Ideally, knockout (via CRISPR) of the genes under study would provide a better model, providing results for chronic rather than acute depletion of the gene of interest. Alternatively, it would be good to see rescue experiments using siRNA resistant expression constructs to rescue the observed phenotypes.

The IFT88 depletion data in figure 4D is not believable, In all other figures (S1A & S1C), blotting for IFT88 reveals a single predominant band at ~88 KDa that disappears in either the ORPK of siRNA conditions. However in the macrophages, the predominant IFT88 band appears to be unchanged, with only a less abundant band disappearing upon 4-OH-T treatment. This would suggest that macrophage may express alternate splice forms of IFT88 that may not contain the floxed exon/s. The qRT-PCR data would seem to suggest this as 4-OH-T treatment only seems to deplete the mRNA by half (less than siRNA treatment in other experiments). shRNA should be performed on macrophages (by lentivirus) to confirm IFT88 isoforms. If macrophage, which are non-ciliated contain an alternate IFT88 isoform, this could potentially provide some novel insight into IFT88 function in a non-cilial context.

Reviewer 2

Advance summary and potential significance to field

In this manuscript McFie et al. investigate the effect of IFT88 absence (knock-out or siRNA) on NFkappaB signalling. They find that IFT88 absence affects NOS and COX signalling, amongst others, but not Jun. They also find that this is the case in non-ciliating cells. Since they observe p65 at the basal body, they propose that this might act as a signalling hub for NFkappaB. They perform RNASeq analysis on IL-1beta induced cells and find that many centrosome/cilium proteins are induced. When three of these proteins are individually targetted, the effect on inflammatory cytokine response varies for diminution to enhancement.
This manuscript is rich in data, data which supports the authors conclusions. The manuscript provides original insight into the relationship between NFkappaB, inflammatory cytokine signalling

© 2020. Published by The Company of Biologists under the terms of the Creative Commons Attribution License (https://creativecommons.org/licenses/by/4.0/).

and the centrosome/cilium that should be in the public domain. The experiments appear technically well executed and data are presented well. The manuscript is vulnerable to criticism of lacking mechanism in that a molecular or biochemical link from IFT88 et al to p65 is not delineated. However, pursuing that link would be another research project and this manuscript does give new information on the link between inflammatory, cytokine, pathways and the centrosome/cilium. This is an area relatively underexplored in the literature.
One minor issue I found was the need to frequently move between main figures and supplementary ones which was awkward.

Comments for the author

I would recommend acceptance with minor revisions given below.
Ln 50 "was both altered during, and 50 potentially influenced, the cell response to the cytokine IL-1β," is a little awkward as altered takes a preposition but influenced does not.
Ln 54 cytokine(s)?
Ln55 The general technique combined with the specific experiment again reads awkwardly.
Ln 102 "body, which is itself formed by modifications to the mature centriole and thus connected to the wider cellular transport cytoskeletal system supporting this role as a transduction hub for cellular signalling" should be comma after centriole. "Wider cellular transport cytoskeletal system is awkward, maybe split the concepts.
Ln 134 "findings- implicating" replace hyphen with comma Ln 136, 137 and others : inconsistency in Fig. or fig.
Ln 178 Use h instead of hr Ln 200-209. The authors comment that the final amount of p65 is similar for the different lines and conditions. They further say the rates are different. However, for the WT conditions, the nuclear levels first dip. Does that affect their rate calculations significantly. Could they comment on this difference?
Ln 282 siRNA in superscript, not SiRNA Ln 321 / Fig 4C. Were the two cells side by side or should there be four separate panels?
Ln 491 delete comma after "Recent studies".
Ln 517 add comma after and Ln 540 "collective" - meaning "set of proteins" - perhaps phrase differently.
Ln 703 units given as U/ml-1 where / is redundant.
Ln 795 best practice to give final concentrations of antibody rather than the dilution from the stock References - many journal titles the upper/lower case needs checking e.g. 877 "Journal of cell science". Perhaps a setting in the referencing software used?

First revision

<u>Author response to reviewers' comments</u>

Reviewer 1
Advance summary and potential significance to field
The manuscript by McFie et al., builds upon previous research indicating that cilia proteins participate in cytokine signalling, namely the response to IL-1B. IN this manuscript the authors perform RNA-seq to identify differential expression of IL-1B responsive genes when IFT88 is transiently knocked down by siRNA. My major concern with this manuscript is that it adds little to the current knowledge of how cilia proteins regulate the IL-1B response. There is no real mechanistic insight provided. In fact, experiments where knockdown of other cilia-associated genes such as Kif3a and TTBK2 give the opposite effect to IFT88 depletion, somewhat confuse the field. The authors speculate that IL-1B signalling is operating at the basal body, but provide no definitive evidence of this.

We concede there is much more to be explored regarding the mechanism for how ciliary proteins regulate the response to cytokine but agree with reviewer 2 that exploring this will be, and indeed is, the subject of an entire new project. This is something we are currently working towards but is beyond this current manuscript. That said in this manuscript we have shown a number of important

© 2020. Published by The Company of Biologists under the terms of the Creative Commons Attribution License (https://creativecommons.org/licenses/by/4.0/).

elements. Whilst the depletion of different ciliary-associated genes have opposing effects, collectively we feel this indicates a non-canonical role for ciliary proteins outside the axoneme.

Firstly, we've shown the effect of IFT88 is associated with a change in NFkB p65 shuttling dynamics. We previously identified a putative functional effect at the level of IKK and as such now propose the regulation is happening either upstream to the centrosome or at the centrosome, potentially directly or indirectly, through the recently identified JLP-supported transport of p65 to the vicinity of the nuclear envelope and potentially nuclear pores beyond. We have now included some of the early data we have regarding JLP, which we also see move to the centrosome during the first 15-30 mins of response to cytokine. Our disruption to P65 dynamic is most evident between 10 and 30 min, JLP accumulation at the centrosome is only apparent from 15 min onwards. We have been, as yet, unable to resolve any differences in centrosomal accumulation of p65 or JLP in IFT88 and TTBK2 disrupted cells. We now would speculate that IFT88 and potentially other ciliary proteins are associated with the process of p65 release and trafficking upstream to the MTOC, but need spatially resolved and sensitive proteomics to test this.

Having identified the IFT88 effect is also cytokine response specific we explored if IFT88 effect has a transcriptional bias, e.g to fast response genes, and showed it is likely the effect is through NFkB bias and the effects are not limited to early response genes.

Importantly, we have shown evidence that the IFT88 regulation does not seem to be an axonemal mechanism and is potentially relevant in non-ciliated macrophages which also express low levels of IFT88.

Reviewer 1 Comments for the author
Some major controls are lacking in the siRNA experiments. Ideally, knockout (via CRISPR) of the genes under study would provide a better model, providing results for chronic rather than acute depletion of the gene of interest. Alternatively, it would be good to see rescue experiments using siRNA resistant expression constructs to rescue the observed phenotypes.

We entirely agree that siRNA controls strengthen findings and have sort to resolve this and opposite effects seen with TTBK2 depletion at the same time.

Firstly, we have added some detail, to make it clear that we used smart pool siRNA (4 different doubled stranded siRNA with seed regions that limit off-target effects). We titrated this down, whilst maintaining effect on targets. In the case of IFT88 we also have presented hypomorph and cre data and thus are confident the effects are through IFT88. We have not yet been able to create and express an siRNA resistant form of IFT88. The use of 4 different siRNA seed regions for each target increased the difficulty of expressing siRNA resistant forms but we have now rescued TTBK2 expression using a cDNA/expression vector (see revised final figure 6E/F). This expression system supports the role for TTBK2 in regulating iNOS expression at baseline and in context of IL-1 induction. TTBK2 overexpression both results in a small increase in iNOS at baseline and an enhancement of IL-1 induced induction.
However, the 'rescue' of expression levels did not reverse the phenotype of depletion. We speculate that this is because the location, interactions and activity of TTBK2, rather than simply its expression levels. It may suggest an indirect effect of depletion of TTBK2 but the fact IFT88, KIF3A, TTBK2 and NPHP4 all affect iNOS induction to us indicate its likely they are all associated with the same complex/mechanism/location of regulation hence our focus on the basal body/centrosome.

The IFT88 depletion data in figure 4D is not believable, In all other figures (S1A & S1C), blotting for IFT88 reveals a single predominant band at ~88 KDa that disappears in either the ORPK of siRNA conditions. However in the macrophages, the predominant IFT88 band appears to be unchanged, with only a less abundant band disappearing upon 4-OH-T treatment.

We have followed up this important point by using an alternative antibody to IFT88 (now shown in figure 5B) which shows 2 bands corresponding to two isoforms of IFT88, which are 4-0H and siRNA sensitive. One of these bands is lost in hypomorphic ORPK condition. Clearly macrophage expression at protein level is lower than in fibroblasts however at mRNA level it is comparable in terms of delta CT to 18S. We have also probed with alternative primers, targeting other regions of IFT88 and

© 2020. Published by The Company of Biologists under the terms of the Creative Commons Attribution License (https://creativecommons.org/licenses/by/4.0/).

again confirmed IFT88 expression whatever site primers are directed to. These primers also indicated a partial removal of IFT88 expression.

This would suggest that macrophage may express alternate splice forms of IFT88 that may not contain the floxed exon/s.

It would appear macrophage might express two isoforms of IFT88, but both are affected by the cre.

The qRT-PCR data would seem to suggest this as 4-OH-T treatment only seems to deplete the mRNA by half (less than siRNA treatment in other experiments). shRNA should be performed on macrophages (by lentivirus) to confirm IFT88 isoforms. If macrophage, which are non-ciliated contain an alternate IFT88 isoform, this could potentially provide some novel insight into IFT88 function in a non-cilial context.

In our opinion our data point outs that bone marrow derived macrophage do appear to express low levels of IFT88. As the reviewer says this may provide novel insight into non-cilial contexts. We have subsequently tried a number of other preparations of 4-OH but no preparation seems to reliably result in a full removal of IFT88 expression at mRNA level. The reductions in IFT88 are however reliable and associated with a phenotype. Our largest variance comes from the iNOS response itself and basal iNOS induction. We are currently working on a better way to remove ciliary proteins that is not associated with baseline effects on the inflammatory markers.

Reviewer 2 Advance summary and potential significance to field
In this manuscript McFie et al. investigate the effect of IFT88 absence (knock-out or siRNA) on NFkappaB signalling. They find that IFT88 absence affects NOS and COX signalling, amongst others, but not Jun. They also find that this is the case in non-ciliating cells. Since they observe p65 at the basal body, they propose that this might act as a signalling hub for NFkappaB. They perform RNASeq analysis on IL-1beta induced cells and find that many centrosome/cilium proteins are induced. When three of these proteins are individually targetted, the effect on inflammatory cytokine response varies for diminution to enhancement.

This manuscript is rich in data, data which supports the authors conclusions. The manuscript provides original insight into the relationship between NFkappaB, inflammatory cytokine signalling and the centrosome/cilium that should be in the public domain. The experiments appear technically well executed and data are presented well. The manuscript is vulnerable to criticism of lacking mechanism in that a molecular or biochemical link from IFT88 et al to p65 is not delineated. However, pursuing that link would be another research project and this manuscript does give new information on the link between inflammatory, cytokine, pathways and the centrosome/cilium. This is an area relatively underexplored in the literature.
One minor issue I found was the need to frequently move between main figures and supplementary ones which was awkward.

Thank you for these comments, we hope the reduction of supplementaries and moving of certain datasets helps with this somewhat.

Reviewer 2 Comments for the author
I would recommend acceptance with minor revisions given below.

Ln 50 "was both altered during, and 50 potentially influenced, the cell response to the cytokine IL-1β," is a little awkward as altered takes a preposition but influenced does not.

Ln 54 cytokine(s)?
Ln55 The general technique combined with the specific experiment again reads awkwardly.

Ln 102 "body, which is itself formed by modifications to the mature centriole and thus connected to the wider cellular transport cytoskeletal system supporting this role as a transduction hub for cellular signalling" should be comma after centriole. "Wider cellular transport cytoskeletal system is awkward, maybe split the concepts.

© 2020. Published by The Company of Biologists under the terms of the Creative Commons Attribution License (https://creativecommons.org/licenses/by/4.0/).

Ln 134 "findings- implicating" replace hyphen with comma

Ln 136, 137 and others : inconsistency in Fig. or fig.

Ln 178 Use h instead of hr

We have addressed these minor comments and thank the reviewer for pointing them out and improving the manuscript. All changes are highlighted in text.

Ln 200-209. The authors comment that the final amount of p65 is similar for the different lines and conditions. They further say the rates are different. However, for the WT conditions, the nuclear levels first dip. Does that affect their rate calculations significantly. Could they comment on this difference?

We calculated rates over 10-30 min period and as such the apparent dip in p65 in WT cells or rather absence of this in IFT88 cells will affect this. The dip whilst slight was robustly seen and may represent a switch from passive P65 nuclear trafficking to active P65 trafficking. We cannot comment further on why this is missing in IFT88 targeted cells but would suggest this may be because of upstream effects on IKK phosphorylation of IKB. It may also be reflective of changes in nuclear entry due to changes in nuclear pores at the centrosome, something we are now exploring further having read Lee et al. 2019.

Ln 282 siRNA in superscript, not SiRNA

Ln 321 / Fig 4C. Were the two cells side by side or should there be four separate panels?

Ln 491 delete comma after "Recent studies".

Ln 517 add comma after and

Ln 540 "collective" - meaning "set of proteins" - perhaps phrase differently.

Ln 703 units given as U/ml-1 where / is redundant.

Ln 795 best practice to give final concentrations of antibody rather than the dilution from the stock

References - many journal titles the upper/lower case needs checking e.g. 877 "Journal of cell science". Perhaps a setting in the referencing software used?

We have addressed these minor comments and thank the reviewer for pointing them out and improving the manuscript. All changes are highlighted in text.

Second decision letter

MS ID#: JOCES/2019/239871
MS TITLE: Ciliary proteins tune NFκB signalling independently of primary cilia to specify the cellular response to inflammatory cytokine
AUTHORS: Megan Mc Fie, Lada Koneva, Isabella Collins, Clarissa R Coveney, Aisling M Clube, Anastasios Chanalaris, Tonia L Vincent, Jelena S Bezbradica, Stephen N Sansom, and Angus KT Wann
ARTICLE TYPE: Research Article

We have now reached a decision on the above manuscript.

To see the reviewers' reports and a copy of this decision letter, please go to:

© 2020. Published by The Company of Biologists under the terms of the Creative Commons Attribution License (https://creativecommons.org/licenses/by/4.0/).

https://submit-jcs.biologists.org and click on the 'Manuscripts with Decisions' queue in the Author Area. (Corresponding author only has access)

As you will see from their reports, both reviewers raise a number of substantial criticisms that prevent me from accepting your paper for publication. The revised manuscript does not overcome their concerns of the level of advance made and as such it is not possible for me to take this further. I am very sorry to give you such disappointing news, but we are currently under great pressure for space and it takes a very enthusiastic recommendation by the referees for a manuscript to be accepted.

I do hope you find the comments of the reviewers helpful in allowing you to revise the manuscript for submission elsewhere, and many thanks for sending your work to Journal of Cell Science.

Reviewer 1

Advance summary and potential significance to field

The manuscript by McFie et al., describes the induction of NFkB signalling components in response to IL-1B in the presence or absence of various cilia components, principally IFT88.

As previously stated in the first review, it is my opinion that this works adds little mechanistic insight into this process compared to previous publications by the senior author. In fact figure 1 of this manuscript replicates the data from the previous manuscript by Wann et al. (Cell Signalling 2014). Specifically in this manuscript they already show that IL-1B signalling (iNOS, COX2, p65) is disrupted in ORPK mouse cells.

Therefore it comes as no surprise that the transcriptional response (as shown by RNA-Seq) is different in cells where IFT88 is depleted/knocked out. This dataset whilst interesting is, as it stands purely descriptive.

As previously stated, I do not feel that, despite the revisions, this paper provides any mechanistic insight and certainly does not provide any clear evidence that the observed effects are working through the centrioles/basal body. Given that this effect of IFT88 depletion on IL-1B signalling has been previously published, I would argue that mechanism is key to making this publication suitable for JCS and not for future studies.

Other work has shown that silencing of Kif3a reduces the IL-1B response (contrary to the findings in this manuscript) arguing that indeed cilia are important for the response (Baek et al., J Neuroinflammation). The fact that IFT88 depletion does not affect p65 centrosomal accumulation may suggest that this effect is not mediated through the centrosome. Alternately, the authors have previously showed that disruption of IFT88 results in disruption of cellular actin, which in turn could affect cell-surface receptor-mediated signalling.

Comments for the author

See comments above.

Rebuttal Letter

MS ID#: JOCES/2019/239871
MS TITLE: Ciliary proteins tune NFκB signalling independently of primary cilia to specify the cellular response to inflammatory cytokine
AUTHORS: Megan Mc Fie, Lada Koneva, Isabella Collins, Clarissa R Coveney, Aisling M Clube, Anastasios Chanalaris, Tonia L Vincent, Jelena S Bezbradica, Stephen N Sansom, and Angus KT Wann
ARTICLE TYPE: Research Article

© 2020. Published by The Company of Biologists under the terms of the Creative Commons Attribution License (https://creativecommons.org/licenses/by/4.0/).

Dear David,

Whilst I completely see the perspective of reviewer 1 might i ask for further consideration?

As reviewer 2 originally stated

The manuscript is vulnerable to criticism of lacking mechanism in that a molecular or biochemical link from IFT88 et al to p65 is not delineated. However, pursuing that link would be another research project and this manuscript does give new information on the link between inflammatory, cytokine, pathways and the centrosome/cilium. This is an area relatively underexplored in the literature. I would recommend acceptance with minor revisions given below.

We would contest the opinion that we at times only replicated data. It was important for us (in figure 1) to check iNOS and COX2 at both mRNA and protein level (previously just protein) to compare ORPK to siRNA of IFT88, which has advantages over the hypomorphic mutation and to build strong platform for IFT88 investigations. We showed that the IFT88-regulation does not effect all responses and is also relevant to TNF. This is important as it supports earlier papers assumptions that effect is at the level of cytoplasmic transduction rather than simply receptor activation. We had previously (2014) shown that IKK activation downstream to receptor and TAK1 (master kinase activated by many receptor systems) was unaffected. The effect therefore was at level of IKK-IKB-p65. We agree our data shows a cytoskeletal level effect (P65 translocation upstream to centrosome) and doesn't prove a centrosomal location for this regulation but both are possible still. We have investigated actin and tubulin in siRNA condition but found no consistent effect when cells are in confluent monolayer (our cells in suspension with ORPK mutation certainly do as previously published). The RNA seq aimed to resolve why some responses and not others were effected and associate global effect with temporal effects at cytosolic signalling level. We felt it was very important to focus on the non-axonemal point (showing in a number of ways that the effect on inflammatory responses did not appear to be dependent or associated with axonemal assembly). We concur that the effects of kif3a seem in contrast to those published. We can only assume this is due to this study using LPS in a neuron or complicated interactions between non-ciliary roles for KIF3A and cell/stimulus type differences. We then followed up with ttbk2 investigations, as this is also centrosomal, was regulated in seq and key to ciliary assembly, rescuing expression as suggested by reviewer 1. The data, collectively with that of IFT88 in macrophages all suggests a non-axonemal role for this set of ciliary proteins in NFkB dependent inflammatory response at the level of IKK-IKB-P65 in the cytosol.

The next step for us is spatial or at least highly sensitive proteomics to understand the transient interactions between ciliary proteins and NFkB potentially through JLP as recently identified. The potential that this regulatory axis is non-axonemal, is not limited to ciliated cells types and is specific to certain responses is key progress in this very novel space, which, we understandably wish to give an excellent platform from which to be read and explored further by the community.

Thank you for your time so far and further consideration on our manuscript.

Kind regards
Angus Wann

Rebuttal Response

MS ID#: JOCES/2019/239871

MS TITLE: Ciliary proteins tune NFκB signaling, independently of primary cilia, to specify the cell inflammatory response

AUTHORS: Megan Mc Fie, Lada Koneva, Isabella Collins, Clarissa R Coveney, Aisling M Clube, Anastasios Chanalaris, Tonia L Vincent, Jelena S Bezbradica, Stephen N Sansom, and Angus KT Wann
ARTICLE TYPE: Research Article

Thank you for your recent letter. I understand how disappointed you must feel.

© 2020. Published by The Company of Biologists under the terms of the Creative Commons Attribution License (https://creativecommons.org/licenses/by/4.0/).

Given the opinions stated by the reviewer, I had no choice but to reject the paper. That said I have had a further detailed look at this and think I might have identified a way forwards.

We are always willing to give authors the chance to defend their manuscripts. In the light of the comments you make in your letter, I have decided to proceed as follows. I would be happy for you to submit a revised version of your manuscript that deals as far as possible with the points raised by the reviewers, together with a detailed rebuttal of any other matter that cannot be settled in the manuscript itself. I may then send the revised version and the rebuttal back to the reviewer but this will depend on my own reading of your rebuttal. If they we convinced by your arguments, then we would be happy to accept the manuscript for publication.

Specifically, I suggest you look to reorganize the manuscript to really emphasize the new findings. One option there might be to remove Figure 1 (or move to a supplemental figure). You should also be clear about the lack of definitive link between your localization data and any mechanistic interpretation invoking centrosomes. You should also address the role of Kif3a in the IL1B response highlighted by the reviewer and the possibility of other effects e.g. through actin.

Second revision

Author response to reviewers' comments

Thank you again for taking the time to go through this again in detail and suggest a way forward.

We have carefully reflected on the reviewer comments and editor suggestions, making (highlighted) changes throughout the manuscript .

The manuscript now conveys the major new findings namely;

1. Depletion of IFT88 inhibits some, but not all, responses to cytokines IL-1 and TNF.
2. IFT88 depletion affects p65 trafficking dynamics.
3. Downstream, 1/3 of the cytokine-regulated transcriptome is selectivity affected by IFT88 depletion due to biase towards NFkB genes over other, unaffected, pathways such as MAPK.
4. IFT88 regulation of NFkB is not reliant upon ciliary axoneme or ciliary kinesin transport.
5. IFT88 regulation of inflammatory response is ‘conserved’ in non-ciliated cell type- the macrophage.
6. IFT88 regulation is cytosolic, upstream to centrosome trafficking of p65.
7. IFT88 positive regulation is joined by positive regulation by NPHP4, dose dependent (depending on expression level) negative regulation by TTBK2 and negative regulation (KIF3A depletion).

To emphasize these points, we have re-organised and re-written sections. Concerning re-organisation, the data concerning the IFT88 ORPK classical inflammatory responses from Figure 1 is moved into supplementary, siRNA remains in main, as new and platform for much of manuscript. Thus Figure 1 is concerned with points one and 2 above.

We have also re-written many sections of the paper to clarify what were the main questions we addressed in this paper and the major findings.

We agree with comments regarding P65 location and have, throughout, refined our interpretation of the likely location of regulation to ‘upstream to centrosome trafficking’ and to make it clear that our data does not resolve this location, through observing p65 at the centrosome/MTOC.

Regarding, KIF3a, we can see this apparent paradox with the 2017 paper in neurons. In our cells (more analogous to a fibroblast than a neuron) we can only speculate we are, by depleting cells of KIF3A, indirectly affecting IFT88 regulation or the role of KIF3A here, is literally in the opposite direction to IFT88 in the cytosolic trafficking of p65. This is clearly speculation; thus we have

© 2020. Published by The Company of Biologists under the terms of the Creative Commons Attribution License (https://creativecommons.org/licenses/by/4.0/).

commented in the discussion that it is likely different our cells use KIF3A differently to a neuron where it is an important axon transport kinesin (outside of cilium) and there may be differences depending on the inflammatory stimulus used.

We have also expanded upon the suggestion regarding effects on actin cytoskeleton. We do have some data regarding basic cytoskeletal (actin and tubulin) structure in our experimental conditions, which suggested, by superficial microscopy analyses, no differences between siCTRL and IFT88 or TTBK2 depleted cells. Indeed, as other signaling pathways appear unaffected we don't think this is a global effect on cytoskeleton, as could be the case in other contexts. However, we have made sure to mention that both IFT88 and TTBK2 are potentially regulating cytoskeletal structure or trafficking - we have no data beyond the p65 shuttling to support this or rule it out.

Thank you for taking the time to look again at our manuscript, to review these arguments and revisions.

Kind regards

Angus Wann

Third decision letter

MS ID#: JOCES/2019/239871

MS TITLE: Ciliary proteins specify the cell inflammatory response by tuning NFκB signaling, independently of primary cilia.

AUTHORS: Megan Mc Fie, Lada Koneva, Isabella Collins, Clarissa R Coveney, Aisling M Clube, Anastasios Chanalaris, Tonia L Vincent, Jelena S Bezbradica, Stephen N Sansom, and Angus KT Wann
ARTICLE TYPE: Research Article

We have now reached a decision on the above manuscript.

To see the reviewer's reports and a copy of this decision letter, please go to: https://submit-jcs.biologists.org and click on the 'Manuscripts with Decisions' queue in the Author Area. (Corresponding author only has access to reviews.)

As you will see, the reviewers gave favourable reports but raised some critical points that will require amendments to your manuscript. I hope that you will be able to carry these out, because I would like to be able to accept your paper.

My decision is based on our editorial thinking outlines below that you will not be able to address these concerns in a reasonable timeframe. I would however like you to make very clear within each figure legend the cell type used in each case. Furthermore, I would ask that you explicitly address the caveats around centrosomal labelling and the conclusions that you can draw there. If you are able to do this then I would expect to able to accept your paper. I would not intend to return this to the reviewer.

We are aware that you may currently be unable to access the lab to undertake experimental revisions. If it would be helpful, we encourage you to contact us to discuss your revision in greater detail. Please send us a point-by-point response indicating where you are able to address concerns raised (either experimentally or by changes to the text) and where you will not be able to do so within the normal timeframe of a revision. We will then provide further guidance. Please also note that we are happy to extend revision timeframes as necessary.

Please ensure that you clearly highlight all changes made in the revised manuscript. Please avoid using 'Tracked changes' in Word files as these are lost in PDF conversion.

© 2020. Published by The Company of Biologists under the terms of the Creative Commons Attribution License (https://creativecommons.org/licenses/by/4.0/).

I should be grateful if you would also provide a point-by-point response detailing how you have dealt with the points raised by the reviewers in the 'Response to Reviewers' box. Please attend to all of the reviewers' comments. If you do not agree with any of their criticisms or suggestions please explain clearly why this is so.

Reviewer 1

Advance summary and potential significance to field

Please see previous reviews

Comments for the author

A major problems with this draft of the manuscript is the constant changing between different cell types (chondrocytes vs macrophages) throughout, especially at the end. Furthermore, the figure legends do not state the cell lines used to generate the data shown.

In the last paragraph of the results section, the authors stated that they were unable to see any differences in centrosomal JLP localisation following ift88 knockdown. They do not show whether ift88 depletion affects p65 centrosomal localisation and in figure 1 I can see no centrosomal p65. These two facts taken together, makes it very hard to argue that the centrosome plays any role without further experiments. It would be good to assess centrosomal p65 by performing centrosomal purifications followed by Western blotting. This is important, especially given that apart from the nuclear p65, the p65 signal is seen throughout the cytosol (figure 6h)
If the authors want to improve on these observations, more details on the localisation would be important - is it at transition zone, distal/subdistal appendages, pericentriolar material, pericentriolar satellites etc.. This is especially important as rabbit polyclonal antibodies have a tendency to light up the centrosome. It would be good to confirm this localisation using a GFP-tagged p65 in live cells to rule out this possibility and partnered with live cell imaging could provide temporal information.

Another important question is whether p65 and IFT88 are localised to the centrosome in macrophages. This is a very important to demonstrate, as if neither are found at this localisation in this non-ciliated cell, the author's speculation that regulation is occurring at the level of the centrosome is difficult to conceive.

Minor points

-Does IFT88 depletion in cells used affect cell cycle and thus degree of ciliation?
-Does knockdown of IFT88 affect the ciliary extension caused by IL-1B and TNFa?
-Need to show graph of cilia length over time to convince reader that early transcriptional responses are temporally distinct from cilia extension.
-Better to use subcellular fractionation in addition to imaging to quantitate nuclear p65 levels by Western blot
- Why in figure S2A is there increased nuclear p65 in the IFT88 siRNA compared to WT at all time points (IL-1B treatment)? This suggests that knockdown of IFT88 enhances p65 nuclear accumulation. This was not observed at the 0 time point for TNFa treatment though, so why the discrepancy in starting levels of nuclear p65?
- IF the manuscript is focussing on IFT88 siRNA, why in figure 1H is the ORPK results shown. Should this not be swapped with the supplemental figure S2A?
-Is the variability in Nos2 expression following tamoxifen treatment due to varied CRE activity?
-The authors state that p65 is frequently located at the centrosome- where is the quantification of this? In figure 1 there appears to be no obvious non-nuclear p65 in a field of ~30-40 cells.

Abstract - line 47 change "a mutation" to "disruption"
Results - line 149 - what cells being used?
Figure 1 legend - does not state cell type Figure 1d - no indication of statistical significance bars/p values (as represented as asterisks in figure 1a for example)

© 2020. Published by The Company of Biologists under the terms of the Creative Commons Attribution License (https://creativecommons.org/licenses/by/4.0/).

Figure 1e - need to split images into constituent channels (red/blue/green)
Figure S2B - please give P value

Third revision

Author response to reviewers' comments

Dear David,

Thank you for the further consideration and giving us the opportunity to make changes to deal with outstanding comments. We have focussed on cell types in legends and caveats to centrosomal labelling as suggested (underlined in bold below) but dealt with as many other comments as possible without new experiments (changes highlighted in revision).

Please find below our point by point comments and revisions below the statement from the reviewer concerned.

Point by Point

- A major problems with this draft of the manuscript is the constant changing between different cell types (chondrocytes vs macrophages) throughout, especially at the end. Furthermore, the figure legends do not state the cell lines used to generate the data shown.

  We have added cell lines to all the legends in the titles.

- In the last paragraph of the results section, the authors stated that they were unable to see any differences in centrosomal JLP localisation following ift88 knockdown. They do not show whether ift88 depletion affects p65 centrosomal localisation and in figure 1 I can see no centrosomal p65. These two facts taken together, makes it very hard to argue that the centrosome plays any role without further experiments.

  We apologise, the writing was ambiguous. We meant to contrast no obvious change in JLP localisation when IFT88 is depleted with the changes to P65 trafficking when IFT88 is depleted, shown in figure 1. P65 is only visible at centrosome against background cytosolic staining towards end of cytosolic translocation over 30 mins from confocal images taken at each timepoint in parallel to those used for whole nucleus quantification.

  We agree with regards to role for the centrosome and again **have sought to focus on upstream to the centrosome,** rather than at the centrosome. The effects of IFT88 are largely on rate of trafficking (via centrosome) to nucleus. **We have made clear the observation of P65 at the centrosome does not indicate the location of regulatory action of IFT88 but only indicates transit through this site (line 405) and addressed directly lines 408-409.**

- It would be good to assess centrosomal p65 by performing centrosomal purifications followed by Western blotting. This is important, especially given that apart from the nuclear p65, the p65 signal is seen throughout the cytosol (figure 6h). If the authors want to improve on these observations, more details on the localisation would be important - is it at transition zone, distal/subdistal appendages, pericentriolar material, pericentriolar satellites etc.. This is especially important as rabbit polyclonal antibodies have a tendency to light up the centrosome. It would be good to confirm this localisation using a GFP-tagged p65 in live cells to rule out this possibility and partnered with live cell imaging could provide temporal information.

  As discussed above we believe focus next would be upstream to the centrosome although it

© 2020. Published by The Company of Biologists under the terms of the Creative Commons Attribution License (https://creativecommons.org/licenses/by/4.0/).

may be interesting to use higher resolution microscopy to look at P65 trafficking at the centrosome and we would be interested to, as described perform purifications at sites including the centrosome/basal body to give context. We absolutely agree on the point regarding live study using GFP. Pilots for these GFP- tagged P65 experiments were underway to check trafficking rates weren't affected with different tags. We are also trying to identify candidate interactions, at early timepoints, attempting to use P65, GFP, JLP, IFT88 and TTBK2, but these studies are again not ready for addition to the manuscript and are part of a more ambitious plan to use spatial proteomics to uncover these regulatory interactions.

We have addressed and discussed (lines 409-411) important caveats with regard to the use of rabbit antibody and centrosomal localisation. We also tried a mouse monoclonal but could not get a clean P65 band in western blot such as to trust IF signal. Importantly whilst P65 is seen regularly at the centrosome it is not always and is not seen once nuclear translocation is complete, supporting a real localisation due to time-dependence to the staining.

- Another important question is whether p65 and IFT88 are localised to the centrosome in macrophages. This is a very important to demonstrate, as if neither are found at this localisation in this non-ciliated cell, the author's speculation that regulation is occurring at the level of the centrosome is difficult to conceive.

  The focus of macrophage experiments was non-ciliary role of IFT88 but it would be interesting to observe P65 translocation here too. Where IFT88 is in macrophage indeed important and we are aiming to approach as above with tagged IFT88 first. Endogenous expression of IFT88 is low in macrophage so this may be even harder to localise and requires overexpression.

Minor points

- -Does IFT88 depletion in cells used affect cell cycle and thus degree of ciliation?

  Cell number is not affected in chondrocyte line in these experimental conditions which are designed to use the confluence at ciliation, just after transfection, to control for cell cycle differences which do occur otherwise. Change to ciliation are shown in Figure 1A but are independent to ki67 status for example.

- -Does knockdown of IFT88 affect the ciliary extension caused by IL-1B and TNFa?

  This paper didn't continue to explore ciliary elongation, the subject of previous papers and work of others but knockdown of ciliary IFT88 does limit elongation in response to IL-1B.

- -Need to show graph of cilia length over time to convince reader that early transcriptional responses are temporally distinct from cilia extension.

  This has been previously published for primary cells (Wann et al. 2013, line 116), elongation is not measurably seen in these cells until at least 12h post cytokine.

- -Better to use subcellular fractionation in addition to imaging to quantitate nuclear p65 levels by Western blot

  Indeed we attempted western blotting of cytosolic and nuclear fractions but did not get as quantitatively robust data over this tight time-course using blotting as we did with imaging and felt the imaging allowed single cell analysis of ciliated vs non-ciliated.

- Why in figure S2A is there increased nuclear p65 in the IFT88 siRNA compared to WT at all time points (IL-1B treatment)? This suggests that knockdown of IFT88 enhances p65 nuclear accumulation. This was not observed at the 0 time point for TNFa treatment though, so why

© 2020. Published by The Company of Biologists under the terms of the Creative Commons Attribution License (https://creativecommons.org/licenses/by/4.0/).

the discrepancy in starting levels of nuclear p65?

This difference is between ORPK and WT but we don't, believe this to be a biological difference as you don't see it with TNF at 0h despite these being effectively identifical. Hence we showed normalised data in main figure. Experiments were run in parallel but not all imaged at the same time, which may have caused differences between IL-1 and TNF which weren't being compared.

- IF the manuscript is focussing on IFT88 siRNA, why in figure 1H is the ORPK results shown. Should this not be swapped with the supplemental figure S2A?

  We have conducted these experiments with IFT88 siRNA but not to the same extent and largely saw same effect as in ORPK.

- -Is the variability in Nos2 expression following tamoxifen treatment due to varied CRE activity?

  No we believe this variability does not correlate to apparent knock-down at least, cre activity is fairly consistent

- -The authors state that p65 is frequently located at the centrosome- where is the quantification of this? In figure 1 there appears to be no obvious non-nuclear p65 in a field of ~30-40 cells.

  Line 457-461 seek to clarify this somewhat but we have not quantified as qualitative observation towards end of translocation in confocal imaging with caveats already discussed.

- Abstract - line 47 change "a mutation" to "disruption"
  changed as suggested

- Results - line 149 - what cells being used?
  clarified line 148-49

- Figure 1 legend - does not state cell type
  Added to title

- Figure 1d - no indication of statistical significance bars/p values (as represented as asterisks in figure 1a for example)
  Lack of significant difference now added to graph.

- Figure S2B - please give P value
  Now Added to legend

Fourth decision letter

MS ID#: JOCES/2019/239871

MS TITLE: Ciliary proteins specify the cell inflammatory response by tuning NFκB signaling, independently of primary cilia.

AUTHORS: Megan Mc Fie, Lada Koneva, Isabella Collins, Clarissa R Coveney, Aisling M Clube, Anastasios Chanalaris, Tonia L Vincent, Jelena S Bezbradica, Stephen N Sansom, and Angus KT Wann
ARTICLE TYPE: Research Article

© 2020. Published by The Company of Biologists under the terms of the Creative Commons Attribution License (https://creativecommons.org/licenses/by/4.0/).

I am happy to tell you that your manuscript has been accepted for publication in Journal of Cell Science, pending standard ethics checks.

© 2020. Published by The Company of Biologists under the terms of the Creative Commons Attribution License (https://creativecommons.org/licenses/by/4.0/).
